# Managing genomic diversity in conservation programs of Chinese domestic chickens

**DOI:** 10.1186/s12711-023-00866-3

**Published:** 2023-12-14

**Authors:** Mengmeng Zhang, Shiwei Wang, Ran Xu, Yijun Liu, Han Zhang, Mengxia Sun, Junyan Wang, Zhexi Liu, Keliang Wu

**Affiliations:** 1 College of Animal Science and Technology, China Agricultural University, Beijing, 100193 People’s Republic of China; 2 Beijing Capital Agribusiness Future Biotechnology Co., Ltd., No. 75 Bingjiaokou Hutong, Beijing, 100088 People’s Republic of China; 3 College of Animal Science, Southwest University, Chongqing, 402460 People’s Republic of China

## Abstract

**Background:**

Effective conservation and utilization of farm animals are fundamental for realizing sustainable increases in food production. In situ and ex situ conservation are the two main strategies that are currently used to protect the genetic integrity of Chinese domestic chicken breeds. However, genomic diversity and population structure have not been compared in these conserved populations.

**Results:**

Three hundred and sixty-one individuals from three Chinese domestic chicken breeds were collected from populations conserved in situ and ex situ and genotyped using genotyping-by-sequencing (GBS). First, we used different parameters based on heterozygosity, genomic inbreeding, and linkage disequilibrium to estimate the genomic diversity of these populations, and applied principal component analysis (PCA), neighbor-joining tree, and ADMIXTURE to analyze population structure. We found that the small ex situ conserved populations, which have been maintained in controlled environments, retained less genetic diversity than the in situ conserved populations. In addition, genetic differentiation was detected between the in situ and ex situ conserved populations of the same breed. Next, we analyzed signatures of selection using three statistical methods (fixation index (F_ST_), nucleotide diversity (*Pi*), and cross-population extended haplotype homozygosity (XP-EHH) to study the genetic footprints that underlie the differentiation between in situ and ex situ conserved populations. We concluded that, in these small populations, differentiation might be caused by genetic drift or by mutations from the original populations. The differentiation observed in the population of Beijing You chicken probably reflects adaptation to environmental changes in temperature and humidity that the animals faced when they were moved from their place of origin to the new site for ex situ conservation.

**Conclusions:**

Conservation programs of three Chinese domestic chicken breeds have maintained their genomic diversity to a sustainable degree. The small ex situ conserved populations, which are maintained in controlled environments, retain less genetic diversity than populations conserved in situ. In addition, the transfer of populations from their place of origin to another site for conservation purposes results in genetic differentiation, which may be caused by genetic drift or adaptation. This study provides a basis for further optimization of in situ and ex situ conservation programs for domestic chicken breeds in China.

**Supplementary Information:**

The online version contains supplementary material available at 10.1186/s12711-023-00866-3.

## Background

Domesticated chickens are one of the most ubiquitous domestic animal species in the world, they are bred for meat and eggs, and also for entertainment. Because of its long history of animal husbandry and diversified geographical conditions, China has a rich diversity of domestic chicken breeds. To date, 107 Chinese breeds have been described [1], among which some have a striking appearance and economically valuable traits. Genetic diversity provides the raw material for breed improvement and for adaptation to changing environments and market demands. Unfortunately, throughout the world the genetic diversity of many species is declining or at risk. Among the domesticated avian species, chickens have by far the largest number of breeds at risk on a global scale [2]. Globally, a large proportion of domestic chicken breeds are becoming extinct or are at a risk of extinction, and this proportion has increased from 24.8 in 2014 to 30.7% in 2018. The majority of these breeds are at risk because a small number of large breeding companies dominate the global market and supply genetics for almost all broiler and egg production chickens in the world. This dominance has resulted in the loss of genetic diversity and in risks to animal welfare [2–4]. In China alone, 21 breeds are at risk, which represents 1/5 of the total number of domestic chicken breeds [1] and which results from the introduction of exotic chicken breeds. Thus, conserving the genomic diversity of domestic chicken breeds is crucial and urgent for the protection and utilization of endangered animal populations.

Effective conservation and use of farm animals are necessary to obtain and maintain sustainable food production. Conservation plans are commonly classified into three categories: in situ conservation, ex situ in vivo conservation, and ex situ in vitro conservation. In vivo methods are primarily used in China for the management of animal genetic resources, including both in situ and ex situ conservation. In situ conservation can best be described as the sustainable breeding of an endangered livestock breed in its normal production environment, or as close to it as practically possible, to conserve genetic diversity over a long period. Ex situ conservation is the preservation of endangered livestock outside of their normal production environment and systems [5, 6]. In China, two national gene banks (National Chicken Genetic Resources in Jiangsu and Zhejiang) and 23 National Conservation Farms have been established. In total, 128 chicken breeds have been conserved at the National Conservation Farms (in situ), and 28 of these are conserved in the National Chicken Genetics Resources Gene Bank (NCGR, Jiangsu) (ex situ) [7]. Comparison of these two in situ and ex situ conservation programs can contribute to a better understanding of their impact on the maintenance of genomic diversity and lead to an increase in their effectiveness. However, few studies have compared the actual efficacy of in situ and ex situ efforts to conserve chickens, although the FAO has recommended that the conservation status of livestock breeds be monitored regularly [8]. Remarkably, for some domestic chicken breeds, the environment and climate differ between in situ and ex situ conditions. Thus, to improve conservation programs, it is important to characterize the adaptation of a given breed from its state of origin to that in national gene banks. A comprehensive knowledge of the genetic diversity within and between breed populations is required to manage animal genetic resources [9]. DNA markers are the most reliable molecular tools for the assessment of genetic diversity [10]. Restriction fragment length polymorphism (RFLP) [11, 12], mitochondrial DNA (mtDNA) [13–17], random amplified polymorphic DNA (RAPD) [18–21], amplified fragment length polymorphism (AFLP) [22–24], Y-chromosome markers [25, 26], variable number of tandem repeats (VNTR) [27], and single nucleotide polymorphisms (SNPs) [10, 28–32] have been the most widely used marker systems. Until relatively recently, conservation programs have been based on pedigree information. The development of high-throughput genotyping techniques has made it possible to obtain large numbers of genomic markers that can be used to correct and reconstruct pedigrees. Genome-wide marker data are also regarded as a useful tool for the maintenance of genetic diversity [33]. Here, we studied three representative Chinese domestic chickens from in situ and ex situ conserved populations using both genomic data and data on their management features to: (i) determine their genomic diversity, (ii) assess the efficacy of ongoing in situ and ex situ conservation efforts, and (iii) detect genomic signatures that result from genetic differentiation between populations that have been managed by two conservation practices, i.e. in situ and ex situ.

## Methods

### Populations

**Fig. 1:**
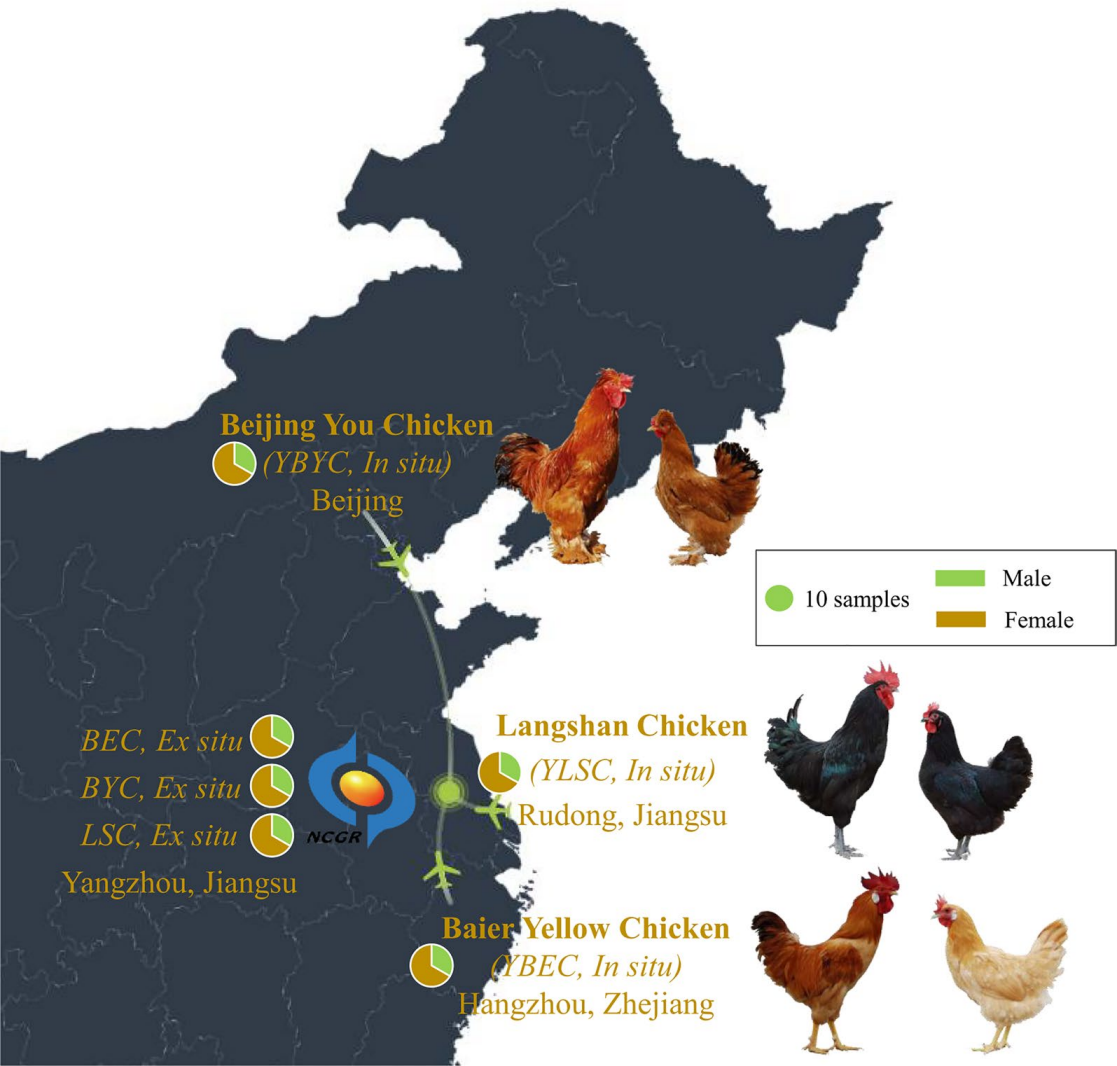
Map of the part of China showing the chicken populations included in this study. BEC, Baier Yellow chicken (ex-situ); YBEC, Baier Yellow chicken (in-situ); BYC, Beijing You chicken (ex-situ); YBYC, Beijing You chicken (in-situ); LSC, Langshan chicken (ex-situ); YLSC, Langshan chicken (in-situ). Male and female specimens are shown for the three breeds. Each subpopulation in the study consisted of 10 males and 20 females (green and brown areas in the pie charts, respectively). Airplane glyphs indicate that individuals from each breed were moved from their original locations (in situ in Beijing, Hangzhou, and Rudong) to Yangzhou for ex situ conservation under the auspices of NCGR (National Chicken Genetic Resources) in Jiangsu

Three hundred and sixty-one individuals from three Chinese domestic chicken breeds were selected from in situ and ex situ conserved populations (120 Beijing You, 120 Baier Yellow, and 121 Langshan). These breeds originate from three different regions in China (see Table 1 and Fig. 1). Of these, 270 chickens (representing three successive conserved generations from an ex situ conserved population) had been genotyped and used in Zhang et al. [34]. In the current study, we used genotyping-by-sequencing (GBS) data to genotype 91 individuals (30 Beijing You,, 30 Baier Yellow, and 31 Langshan individuals) that are part of an in situ conservation program. Blood samples were collected from the wing vein and stored at − 20 °C. We used the Qiagen DNeasy Tissue kit (Qiagen, Germany) to extract genomic DNA from blood, and verified the integrity and purity of DNA by agarose gel electrophoresis and optical density (A_260_/A_280_ ratio). Three μg of high-quality DNA were used to construct sequencing libraries for each sample.

**Table 1:** Pedigree information for the in-situ and ex-situ conserved chicken populations

Breeds	In-situ								Ex-situ							
	Conservation first generation	Conservation scale		Samples		Sampling collection location	Sampling collection time	Code	Conservation first generation	Conservation scale		Samples		Sampling collection location	Sampling collection time	Code
		Sire	Dam	Sire	Dam					Sire	Dam	Sire	Dam			
Baier Yellow Chicken	1982	≥ 30	≥ 300	10	20	Zhejiang	2018	YBEC	1998	30	300	10	20	NCGR (Jiangsu)	2007	BEC07
												10	20		2010	BEC10
												10	20		2015	BEC15
Beijing You Chicken	1972	≥ 30	≥ 300	10	20	Beijing	2018	YBYC	1976	30	300	10	20	NCGR (Jiangsu)	2007	BYC07
												10	20		2010	BYC10
												10	20		2015	BYC15
Langshan Chicken	1959	≥ 30	≥ 300	10	20	Jiangsu	2018	YLSC	1998	30	300	10	20	NCGR (Jiangsu)	2010	LSC10
												10	20		2012	LSC12
												10	20		2015	LSC15

*NCGR* National Chickens Genetic Resources (Jiangsu)

*YBEC* Baier Yellow Chicken in in-situ, *YBYC* Beijing You Chicken in in-situ, YLSC, Langshan Chicken in in-situ, *BEC07* Baier Yellow Chicken in ex-situ conserved in 2007, *BEC10* Baier Yellow Chicken in ex-situ conserved in 2010, *BEC15* Baier Yellow Chicken in ex-situ conserved in 2015; *BYC07* Beijing You Chicken in *ex-situ* conserved in 2007; *BYC10* Beijing You Chicken in ex-situ conserved in 2010, *BYC15* Beijing You Chicken in ex-situ conserved in 2015, *LSC10* Langshan Chicken in ex-situ conserved in 2010; *LSC12* Langshan Chicken in ex-situ conserved in 2012, *LSC15* Langshan Chicken in ex-situ conserved in 2015

Altogether 361 individuals were analyzed, defining 12 subpopulations (see Table 1). In each breed, the four subpopulations corresponded to in situ samples and three cohorts of ex situ samples (Table 1).

It should be noted that the mating systems used in the two in situ and ex situ conservation systems differ; in situ conservation uses R:R random mating with random selection, and ex situ uses R:F random mating within families. In R:R, taking 400 hens from 40 breeding families as an example, the rooster from family 1 randomly mates with 10 out of 390 hens from families 2 to 40, the rooster from family 2 randomly mates with 10 out of 390 hens from families 1, 3 to 40, and so on. In R:F, random mating is carried out within each of multiple family groups with equal offspring contribution, with each family group consisting of 1 rooster and 10 hens for breeding. Each hen from the parental generation is required to contribute one offspring for breeding, while any hen without offspring is randomly replaced by an offspring from other hens in the same family group. In addition, compared to the ex situ chickens, the in situ conserved chickens have been subjected to conservation processes for a longer time and have a larger population size.

### Genotyping

We processed the samples as described in Zhang et al. [34]. After double digestion with *MseI* and *HaeIII*, all DNA samples were genotyped by high-throughput sequencing at average depth of 11.28 × using an Illumina HiSeq 4000 sequencer (Illumina, San Diego, CA, USA) and the protocol provided by the manufacturer. To improve mapping, in-house scripts were used to remove low-quality reads from the dataset. Reads were excluded if they (i) contained adapter sequences, (ii) if more than 10% of the nucleotides were unidentified (N), or (iii) if more than 50% of the bases had low phred quality scores ($<$ 5). The remaining high-quality paired-end reads were mapped to the *Gallus gallus* 5.0 reference genome using the Burrows-Wheeler Alignment tool (BWA) (v0.7.8) [35] with default parameters. PCR duplicates were removed using the SAMtools rmdup (v1.3.1) software [36].

The aligned BAM files for the 361 chickens were used to detect variants at the population scale using the SAMtools suite (v1.3.1), including BCFtools, with parameters as described in Zhang et al. [34]. Single nucleotide variants (SNVs) within 5 bp of an insertion/deletion (INDEL) polymorphism were removed. SNPs and INDEL were annotated with the ANNOVAR v2013-08-23 software (ANNOVAR, RRID:SCR 012821) [37], using gene annotations from the Ensembl database (https://asia.ensembl.org/). For the annotation step, SNPs and INDEL were classified into eight categories based on genomic locations, including exonic regions (synonymous, nonsynonymous, stop gain, and stop loss), splicing sites, intronic regions, 5’ and 3’ UTR, upstream and downstream regions, and intergenic regions. The dbSNP database [38] was used to identify novel genetic variants.

SNPs with an unknown chromosome location were removed. Data were also excluded for: (i) individuals with missing genotype data for more than 5% of the typed SNPs (call rate ≤ 0.95), (ii) variants with missing call rates ≥ 0.01, (iii) SNPs with very low minor allele frequencies (MAF ≥ 0.01), or (iv) SNPs with frequencies that deviated significantly from Hardy–Weinberg equilibrium (P-value > 10^–6^). Removal of low-quality SNPs helped to avoid false-positives and also enhanced the ability to identify loci associated with traits and estimate effective genomic diversity.

### Population structure analysis

The neighbor-joining (NJ) tree was constructed using the neighbor-joining method in the MEGA v7.0 software [39] and was visualized with the graphical viewer FigTree v1.4.4 [40]. Population stratification was analyzed by complete linkage clustering of individuals using genome-wide SNP data in the PLINK software [41]. A principal component analysis (PCA) [42] was conducted using PLINK, and scatter plots were generated using a custom R v3.5.3 script [43]. Population structure was analyzed using the ADMIXTURE v1.3.0 software [44], which applies a likelihood model to large whole-genome SNP genotype datasets. The number of populations (K) was varied from K = 2 to 9 to obtain the maximum likelihood estimates for inference of population structure. Cross-validation was performed to provide a low cross-validation error and define the optimal K value. The standard errors of the parameters were estimated using 1000 bootstrap replicates. The cross-validation plot was generated using a R v3.5.3 script [43].

### Assessment of the genomic diversity within each population

Allelic richness (*Ar*), proportion of polymorphic markers (*Pn*), expected heterozygosity (*He*), and observed heterozygosity (*Ho*) were used to investigate genome-wide genomic diversity within each of the 12 subpopulations (see Table 1). Allelic richness was calculated using the ADZE v.1.0 program [45] and *Pn*, *He*, and *Ho* were calculated using the PLINK v1.9 software [41].

### Estimation of the inbreeding coefficient (*F*)

Two metrics were used to estimate levels of inbreeding in the conserved chicken populations: *F*_ES_ based on the mating system and *F*_ROH_ based on runs of homozygosity (ROH).

The *F*_ES_ inbreeding coefficient was predicted from the number of parents and the breeding system. The relative change in average inbreeding (∆F) was obtained by linear regression of the average annual inbreeding coefficient over time, Ft = 1-(1-∆F)^t^, where t represents the generation. The increase in hypothetical inbreeding (∆F) differs for different conservation retention modes. For random mating, random selection$\Delta {\text{F}}=\frac{1}{8Nm}+\frac{1}{8Nf}$, and for random mating within families$, \Delta {\text{F}}=\frac{3}{32Nm}+\frac{1}{32Nf}$, where $Nf$ and $Nm$ represent the numbers of dams and sires, respectively.

The *F*_ROH_ statistic, introduced by McQuillan et al. [46], was calculated as follows: *F*_ROH_ = L_ROH_/L_AUT_, where L_ROH_ is the total length of all ROH in the genome of an individual, and L_AUT_ is the specific length of the autosomal genome covered by SNPs.

### Calculation of the nucleotide diversity

The nucleotide diversity (π) for each population was calculated using VCFtools v0.1.14 program [47], based on whole-genome SNPs.

### Linkage disequilibrium decay

Genome-wide linkage disequilibrium (LD) was evaluated between in situ and ex situ groups. The average LD of a pair of SNPs in a 300-kb sliding window was estimated using the Haploview software [48], and the LD decay curves were generated using a R v.3.5.3 script [43] and Adobe Illustrator CC 2018.

### Estimation of population differentiation based on the fixation index *FST*

The fixation index (*F*_ST_), which is a measure of population differentiation and population structure [49], was estimated using the VCFtools v0.1.14 software [47] by setting a 100-kb window size and 10-kb step size.

### Effective population size

We used the NeEstimator v.2.01 software [50] to implement the LD approach of Waples and Do [51] to estimate effective population size (Ne). The estimates of Ne for each subpopulation were calculated as the average of the estimates for the macrochromosomes [*Gallus gallus* chromosome 1 (GGA1) to GGA5] [52].

### Runs of homozygosity

To investigate recent inbreeding and the distribution of homozygosity, we identified the runs of homozygosity (ROH) based on autosomal SNPs using the PLINK v1.9 software [41]. The analysis was conducted using the default parameter *-homozygosity* and setting the following criteria: (i) a sliding window of 50 SNPs across the genome, (ii) one heterozygous and five missing calls were allowed per window to account for genotyping errors, (iii) the minimum number of consecutive SNPs included in a run of homozygosity was set to 50 and the minimum length for a run was set to 100 kb, (iv) the required minimum SNP density to define a run was 1 SNP per 50 kb, and (v) the maximum distance between two consecutive SNPs in a run was 1000 kb [34].

Differences in genome-wide homozygosity between in situ and ex situ populations were tested for statistical significance with three measures: numbers of runs of homozygosity (NSEG), total length of runs (KB), and average length of runs (KBAVG).

### Analysis of signatures of selection

To analyze the genetic mechanisms that underlie adaptation in the in situ and ex situ conserved populations, we used multiple statistical tests to identify genomic regions harboring footprints of positive selection between the groups, i.e. *F*_ST_ [53–55], nucleotide diversity (*Pi*) [56, 57], and cross-population extended haplotype homozygosity (XP-EHH) [58]. A sliding window approach (100-kb windows sliding in 10-kb steps) [7, 59, 60] was applied to quantify the levels of polymorphism, using pairwise nucleotide variation as a measure of variability (θπ) and genetic differentiation (*F*_ST_) between populations. Genomic signatures with significantly high *F*_ST_ values corresponding to the top 5% of values, and θπ ratios in the top 5% of values (θπ, in situ/θπ, ex situ) were classified as extensively diversified. XP-EHH scores were calculated using the Selscan program [61] with default parameters to compare whole-genome SNPs in all three chicken breeds between in situ and ex situ conserved populations. The scores for each SNP were then frequency-normalized over all the chromosomes using the script norm, provided with Selscan.

### Genome annotation and functional enrichment analysis

We used the Ensembl *Gallus gallus* BioMart webtools to retrieve the genes that were associated with the selected genomic regions identified using the methods described above. The retrieved regions were compared to the Animal QTL Database [62] (http://www.animalgenome.org/QTLdb) to identify candidate regions or genes associated with interesting phenotypic or economic traits. Functional enrichment analyses for gene ontology (GO) terms and Kyoto Encyclopedia of Genes and Genomes (KEGG) pathways were performed using the R “clusterprofiler” package [63]. All chicken genes that were annotated in Ensembl were used as a background set. P values (i.e., EASEscore), that indicated that the overlap between various gene sets was significant, were calculated using the Benjamini-corrected modified Fisher’s exact test. Only terms with a P value lower than 0.05 were considered as significant.

## Results

### Genome sequencing and identification of variants

To detect genome-wide variation in the three Chinese chicken breeds that have been conserved in situ, we genotyped 91 individuals using GBS (Fig. 1). Alignment of 79.95 Gb of sequence data against the *Gallus gallus* 5.0 reference genome yielded an average read depth of 6.84 (see Additional file 1: Table S1). These data combined with the genomic data obtained from the same three breeds conserved in the ex situ programs [34], 5,070,414 variants were identified, including 4,709,112 SNPs and 361,302 short INDEL, which were evenly distributed along the genome (see Additional file 2: Fig. S1a and Additional file 3: Fig. S2). 31.58% of these SNPs were novel and were not present in the dbSNP database at NCBI (see Additional file 4: Table S2 and Additional file 2: Fig. S1b). After removal of the variants that did not meet the quality criteria for MAF and Hardy–Weinberg equilibrium (see above), 1,518,758 SNPs remained for further analysis.

### Population structure analysis

**Fig. 2:**
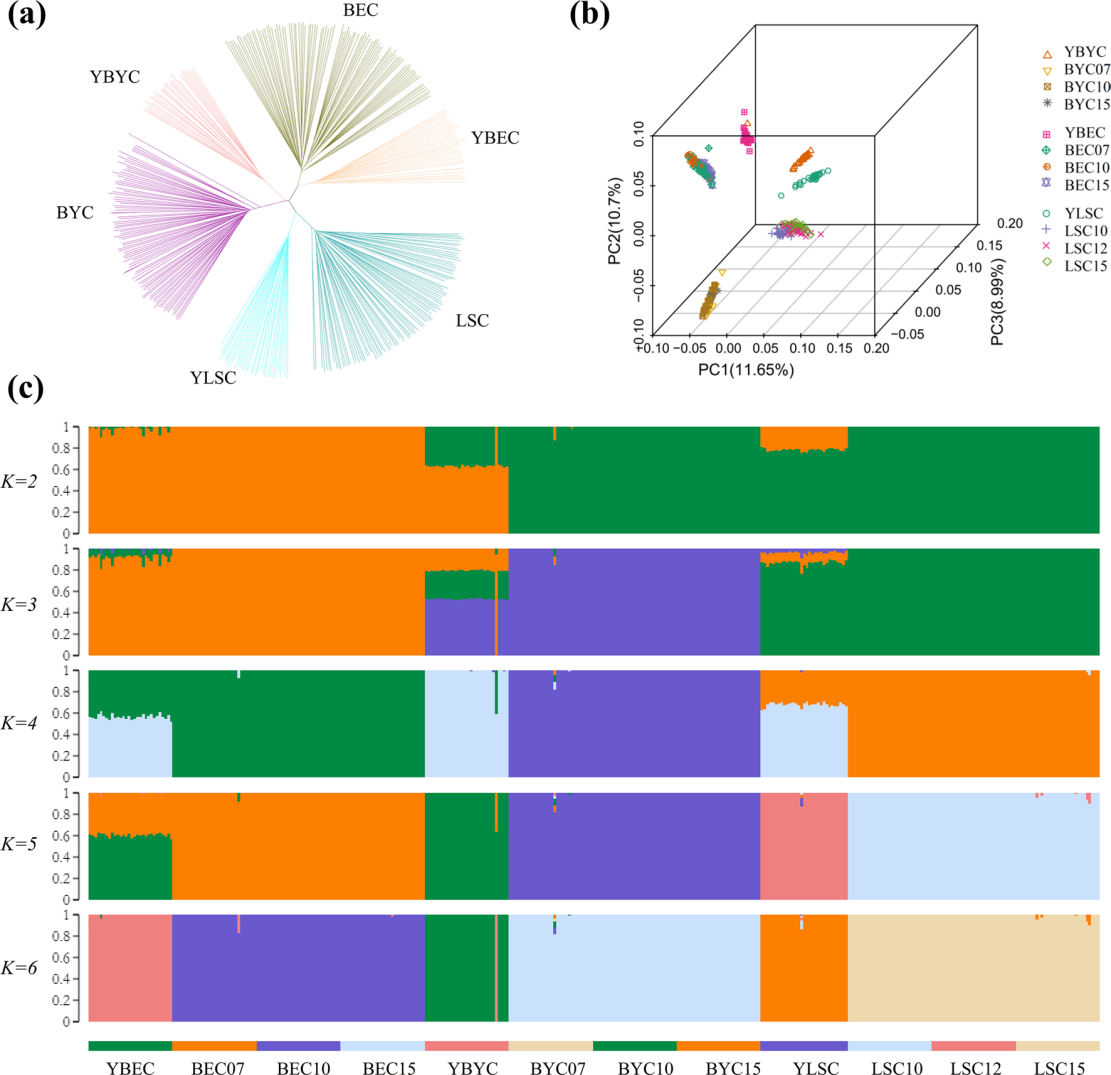
Population genetic structure. **a** Neighbor-joining tree constructed using SNP data from ex situ and in situ conserved populations of the three chicken breeds. **b** PCA analysis of subpopulations. The first three principal components are shown, and the subpopulations are color-coded according to the key to the right. **c** Inferred population genetic structure using the maximum-likelihood method under a model with ancestral components varying from K = 2 to 6

To investigate the phylogenetic relationships and population structure among the 361 chickens, we constructed a neighbor-joining tree using a pairwise genetic distance matrix (Fig. 2a) and performed PCA based on the variance-standardized genotype relationship matrix (Fig. 2b). The neighbor-joining tree suggests that the samples from the six major clusters correspond to the three Chinese domestic chicken breeds, with further subdivision of each breed into in-situ and ex-situ populations. This pattern was further confirmed by PCA. The first principal component (PC1, variance explained = 11.65%) successfully separated the Langshan chicken breed from the other groups. The second principal component (variance explained = 10.7%) separated all the populations in the three chicken breeds (see Additional file 5: Fig. S3). Notably, the PCA separated the in situ and ex situ conserved populations, especially for the Langshan chicken and Beijing You chicken (see Additional file 5: Fig. S3). To better understand population ancestry, we used ADMIXTURE to estimate the number of ancestral populations [44] and allowed the population number (K) to vary from 2 to 9. The minimum estimated cross-validation error occurred at K = 6 (see Additional file 6: Fig. S4). These results suggest that the three Chinese domestic chicken breeds studied here have distinct genetic backgrounds and that their in-situ and ex-situ conserved populations differ, which is consistent with the results from the NJ tree and principal components analyses. The likelihood model based on K = 6 resolves the three Chinese domestic chicken populations into six genetic clusters (Fig. 2c). One individual from the in-situ conserved population of Beijing You chickens had a genetic background that was distinct from the other individuals in this population, based on the NJ tree, PCA, and ADMIXTURE results. Thus, we removed this individual from subsequent analyses.

### Genomic diversity assessment

**Fig. 3:**
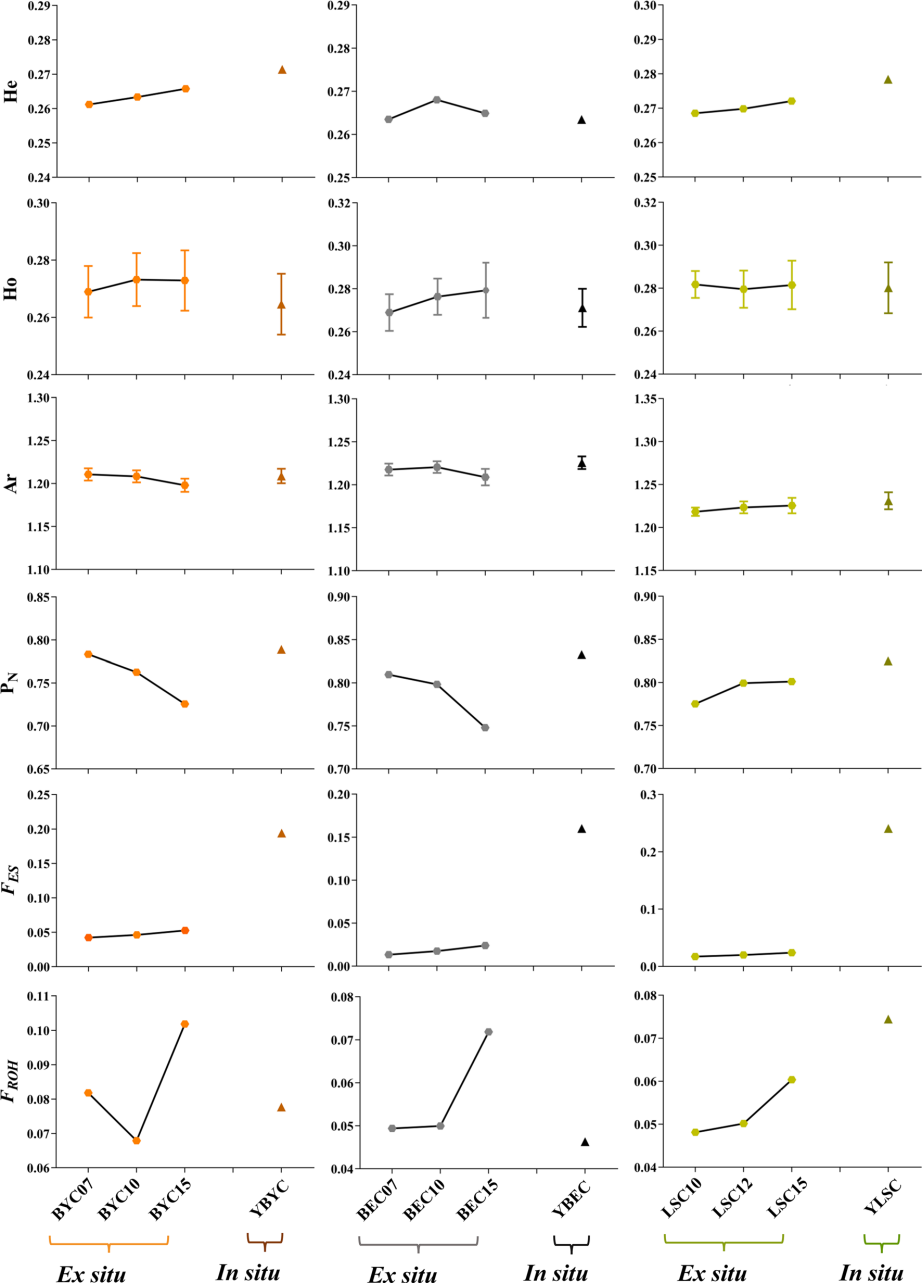
Analysis of genomic diversity between in situ and ex situ populations within breeds. *Ho* observed heterozygosity, *He* expected heterozygosity, *P*_*N*_ proportion of polymorphic markers, *A*_*R*_ allelic richness, *F*_ROH_ inbreeding coefficients based on ROH, *F*_ES_ inbreeding coefficient based on pedigree

Analyses of the *Ho*, *He*, *A*_*R*_, *P*_*N*_ parameters and inbreeding coefficient (*F*) for the six sub-populations are presented in Fig. 3 and Table 2. The genomic diversity in the in situ conserved populations was higher than in the ex situ conserved populations. The *Ho* and *He* were similar for all three breeds and for both the in situ and ex situ conserved populations. For example, changes in genetic diversity between the in situ conserved population of the Beijing You chicken (YBYC, *Ho* = 0.2646, *He* = 0.2714) and the ex situ conserved population (BYC15, *Ho* = 0.2729, *He* = 0.2658) were smaller than 5%. In contrast, *A*_*R*_ and *P*_*N*_ for the in situ conserved population (*A*_*R*_ = 1.209, *P*_*N*_ = 0.7891) were higher than for the ex situ conserved population (*A*_*R*_ = 1.198, *P*_*N*_ = 0.7258).

**Table 2:** Genomic diversity parameters for the three domestic chicken populations

Populations	Ho	He	P_N_ (%)	A_R_	F_ES_	F_ROH_
BEC07	0.2690	0.2635	0.8098	1.218	0.0135	0.0494
BEC10	0.2764	0.2681	0.7983	1.221	0.0175	0.0500
BEC15	0.2793	0.2649	0.7481	1.209	0.0241	0.0719
YBEC	0.2711	0.2635	0.8327	1.226	0.1602	0.0463
BYC07	0.2690	0.2612	0.7833	1.211	0.0424	0.0818
BYC10	0.2732	0.2634	0.7627	1.208	0.0463	0.0679
BYC15	0.2729	0.2658	0.7258	1.198	0.0528	0.0958
YBYC	0.2646	0.2714	0.7891	1.209	0.1942	0.0777
LSC10	0.2818	0.2686	0.7753	1.218	0.0175	0.0481
LSC12	0.2796	0.2699	0.7995	1.223	0.0201	0.0502
LSC15	0.2815	0.2721	0.8013	1.226	0.0241	0.0604
YLSC	0.2802	0.2784	0.8251	1.231	0.2410	0.0745

*He* Expected heterozygosity, *Ho* Observed heterozygosity, *P*_*N*_ Proportion of polymorphic SNPs, *A*_*R*_ Allelic richness, *F*_ES_ inbreeding coefficient based on pedigree; *F*_ROH_ inbreeding coefficient based on the runs of homozygosity

### Estimation of inbreeding coefficients

To estimate the degree of inbreeding in the in situ and ex situ conserved populations, we calculated *F*_ES_ and *F*_ROH_ in each subpopulation. As expected, the *F*_*ES*_ values increased when the conservation procedures were maintained. This trend is also observed in the comparison of *F*_ES_ in in situ vs. ex situ conserved chicken populations. Conservation practices have been applied for a longer period (conservation time; CT) for the in situ population than the ex situ population, and the *F*_ES_ values for the in situ population are correspondingly higher.

Since* F*_ROH_ is more efficient for detecting both rare and common variants [64, 65], we focused on this measurement in subsequent analyses. It was relatively low, ranging from 0.0463 to 0.0958, and except for the Langshan chickens, *F*_ROH_ for the in situ conserved populations was lower than that for the ex situ populations. This difference may be caused by the small size of the Langshan chicken in situ conserved population and its long conservation time (CT = 60 years). The comparison of the inbreeding coefficients for the current generation of all three chicken breeds is presented in Fig. 3 and Table 2.

### Calculation of the nucleotide diversity

**Fig. 4:**
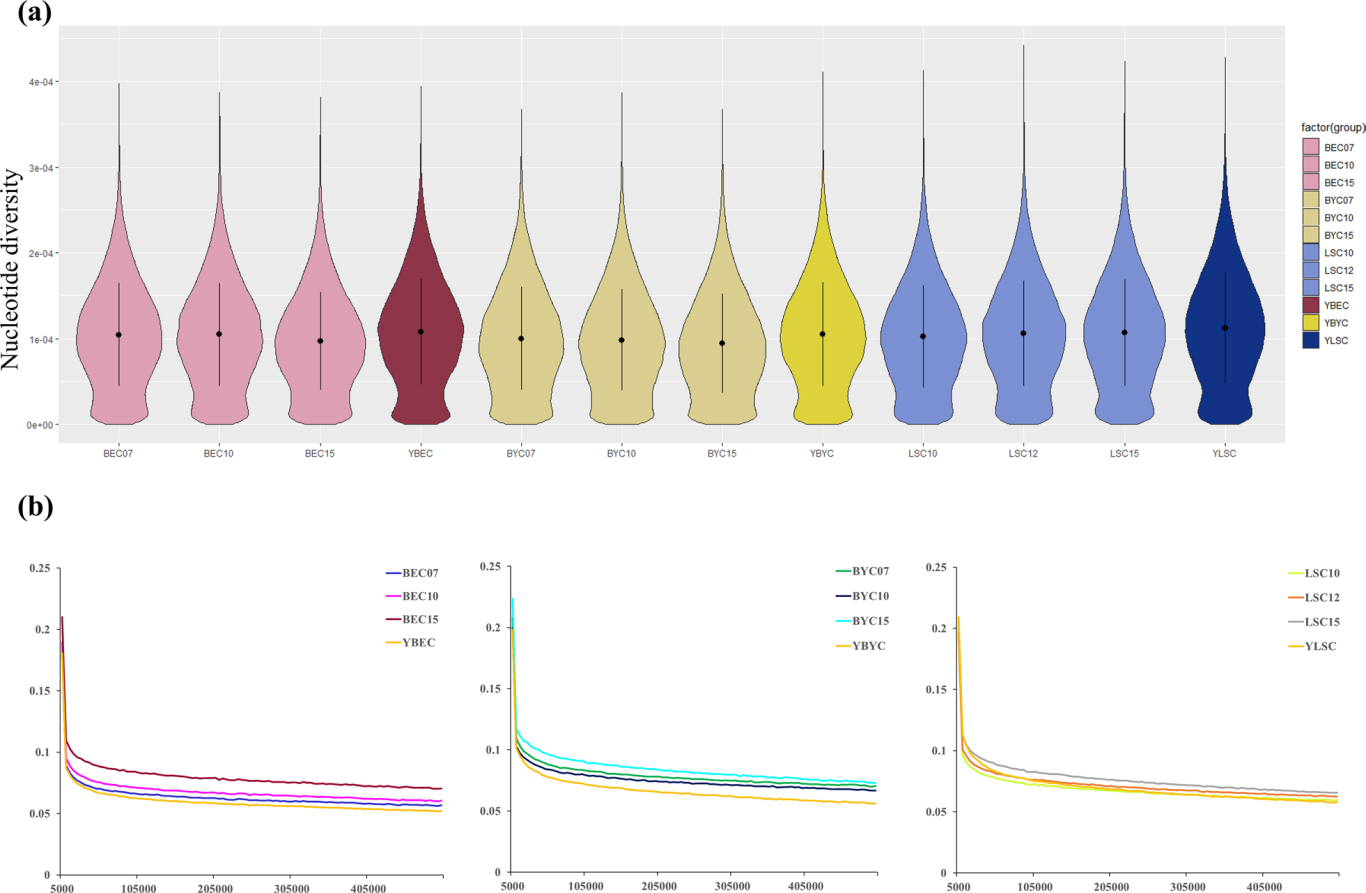
**a** Estimates of population nucleotide diversity (Pi-LSC10 = 0.000102248, LSC12 = 0.000105831, LSC15 = 0.000106998, YBYC = 0.000105085, BYC07 = 0.000100251, BYC10 = 9.84366e−05, BYC15 = 9.46419e−05, YBEC = 0.000107512, BEC07 = 0.000104584, BEC10 = 0.000104865, BEC15 = 9.71941e−05). **b** LD decay determined by squared correlations of allele frequencies (*r*^*2*^) against the distance between polymorphic sites

The results of the *Pi* for the three breeds are shown in Fig. 4a. The Langshan chicken (in-situ) (*Pi* = 0.000112582) had the highest average nucleotide diversity among the 12 subpopulations, followed in descending order by YBEC, LSC15, LSC12, YBYC, BEC10, BEC07, LSC10, BYC07, BYC10, BEC15, and BYC15. For all three chicken breeds, *Pi* was markedly higher in the in situ conserved populations than in the ex situ conserved populations, and highly significant differences (P < 0.001) were observed between populations within breeds.

### Linkage disequilibrium decay

Differences in LD decay between the in situ and ex situ conserved populations are shown in Fig. 4b. The highest maximum average LD (*r*^*2*^ = 0.2235) was observed in the Beijing You chicken population (BYC15), and the lowest (*r*^*2*^ = 0.1806) in the Baier Yellow chicken population (YBEC). Compared to the current generation of the ex situ conserved populations (BYC15, BEC15, and LSC15), the maximum average LD values were lower in the in situ conserved Beijing You chicken and Baier Yellow chicken populations, while higher values were observed in the Langshan chicken population. This may indicate that YBYC and YLSC have greater genetic diversity than BYC15 and LSC15. As expected, LD declined as the physical distance increased between pairwise SNPs. As shown in Fig. 4b, LD decay in the in situ conserved populations declined markedly compared with the ex situ populations for Beijing You chicken and Baier Yellow chicken. In contrast, LD decay was similar in the in situ and ex situ conserved populations for Langshan chickens. Using the Beijing You chickens as an example, *r*^*2*^ decreased by half (from 0.1982 to 0.0991) within a 11.84-kb region in the in situ conserved group, while LD decayed by half within a 14.68-kb region in the ex situ conserved population (BYC15).

### Estimation of population differentiation using *FST*

**Fig. 5:**
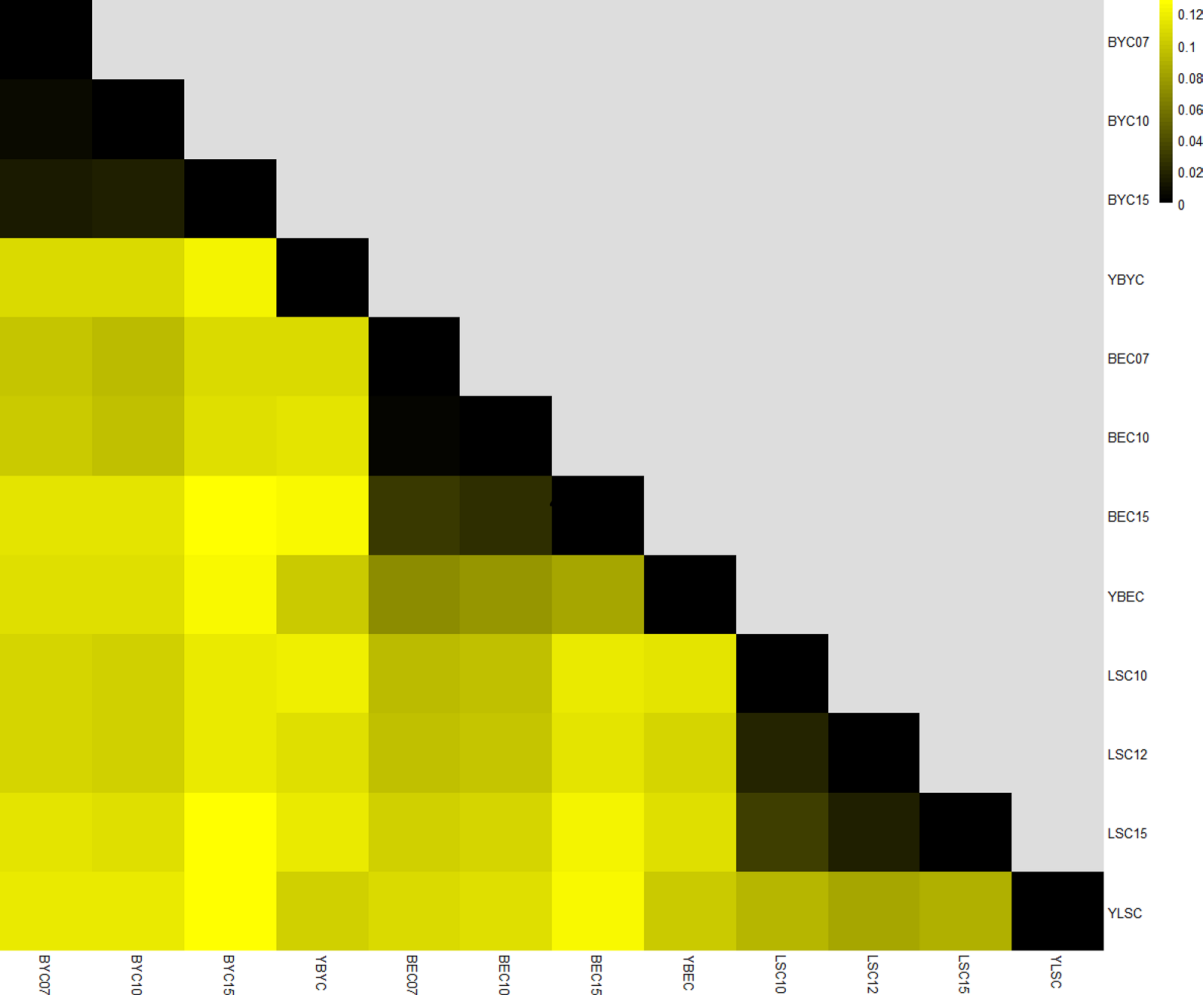
Matrix showing pairwise differentiation estimates (*F*_ST_) between in situ and ex situ conserved populations

To estimate population differentiation, we calculated the pairwise *F*_ST_ values between the sub-populations (see Additional file 7: Table S3), which ranged from 0.004826 to 0.1508. *F*_ST_ values for all pair-wise comparisons are shown in Fig. 5. For all three breeds, *F*_ST_ values over three successive generations were lower than 0.05. This result indicates that no or little genetic differentiation has occurred in the conserved populations from one generation to the next. Significant or moderate genetic differentiation is observed between breeds, and the maximum *F*_ST_ value was calculated between LSC15 and BYC15 (*F*_ST_ = 0.1508). Notably, *F*_ST_ values between the in situ and ex situ conserved populations for all three breeds were higher than 0.05. In the case of the Beijing You chicken, *F*_ST_ values increased with the duration of the conservation program, and the maximum *F*_ST_ value (0.1379) was found between BYC15 and YBYC. Overall, moderate genetic differentiation has occurred in the in situ and ex situ conserved populations for the three chicken breeds.

### Effective population size (Ne)

**Fig. 6:**
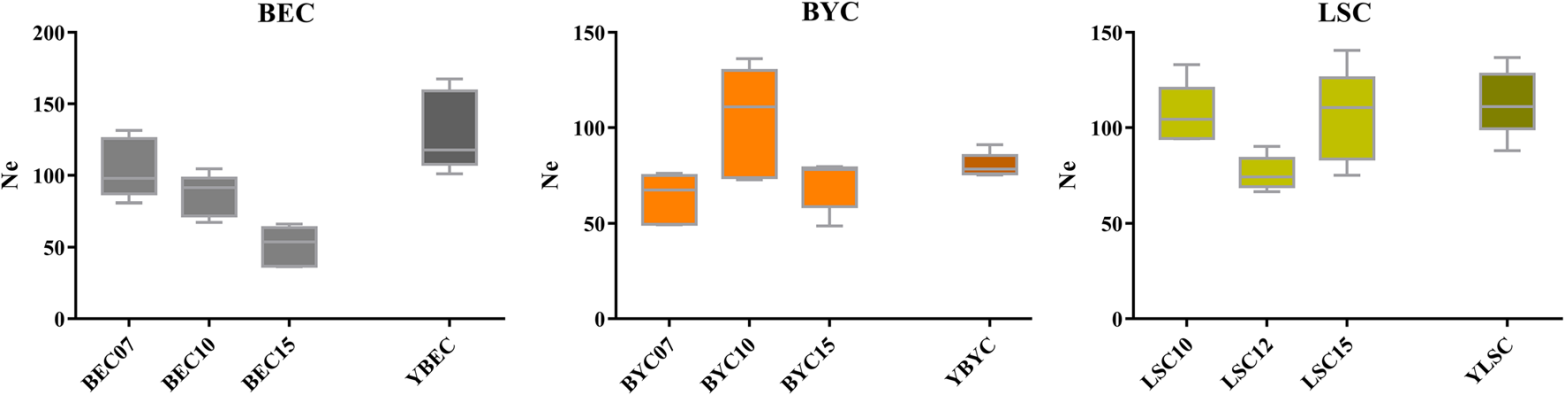
Boxplots showing effective population sizes of the in situ and ex situ conserved populations for each breed

In order to estimate the current Ne for these conserved Chinese domestic chicken breeds, we used NeEstimator v2 [50], which applies a method based on LD to calculate Ne using whole-genome SNPs. Effective population size was estimated for the autosomes GGA1 to GGA28 (see Additional file 8: Table S4) and ranged from 2.7 to 167.4, with a mean of 43.81. Given the huge differences in recombination rate, using LD decay as a function of physical distance logically provides very different Ne estimates. Among the macro-chromosomes (GGA1 to GGA5), BEC15 exhibited the smallest estimated Ne (50.96), suggesting that BEC15 is a limited pool of individuals, whereas YBEC had the largest value (130.28), suggesting much higher genetic diversity. Importantly, Ne in the in situ conserved populations was larger than the in current generations of the ex situ conserved populations (Fig. 6).

### Runs of homozygosity

The abundance and genomic distribution of ROH provide information about the demographic history of a livestock species. ROH were identified in the genomes of all in situ and ex situ conserved populations (see Additional file 9: Table S5). A genome-wide survey for autozygosity was conducted to identify regions with signatures of selection that reflect ancient or recent inbreeding effects. The estimates of *F*_ROH_ were maximum for the ex situ conserved Beijing You chicken population. In contrast, the minimum values occurred in the in situ conserved Baier Yellow chicken population (Table 2). BYC15, the current generation in the ex situ conserved population, had the highest level of inbreeding (0.1018). As expected, YBYC in the in situ conservation population had a lower level of inbreeding (0.0777) than BYC15. YBEC had the lowest level of inbreeding (0.0463) among all the populations. However, within the Langshan chicken breed, YLSC (*F*_ROH_ = 0.0745) had a higher level of inbreeding than LSC15 (*F*_ROH_ = 0.0604).

**Fig. 7:**
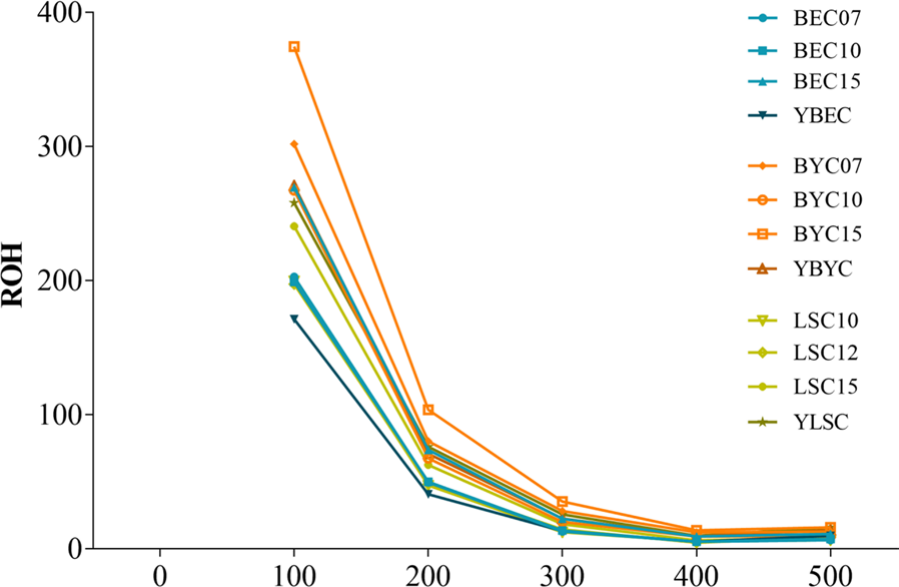
Number of runs of homozygosity (ROH) as a function of run size in kb

Then, we assessed all the ROH to determine whether any populations exhibited evidence of recent inbreeding. For BYC and BEC, the ex situ conserved populations had longer ROH and lower genomic diversity than the in situ conserved populations (Fig. 7a). In contrast, the in situ conserved LSC population had a higher level of inbreeding than the ex situ conserved LSC population. We also mapped ROH to the genome, and found that the homozygosity segments in the in situ vs. the ex situ conserved populations were distributed differently (see Additional file 10: Fig. S5).

### Adaptation analysis

**Fig. 8:**
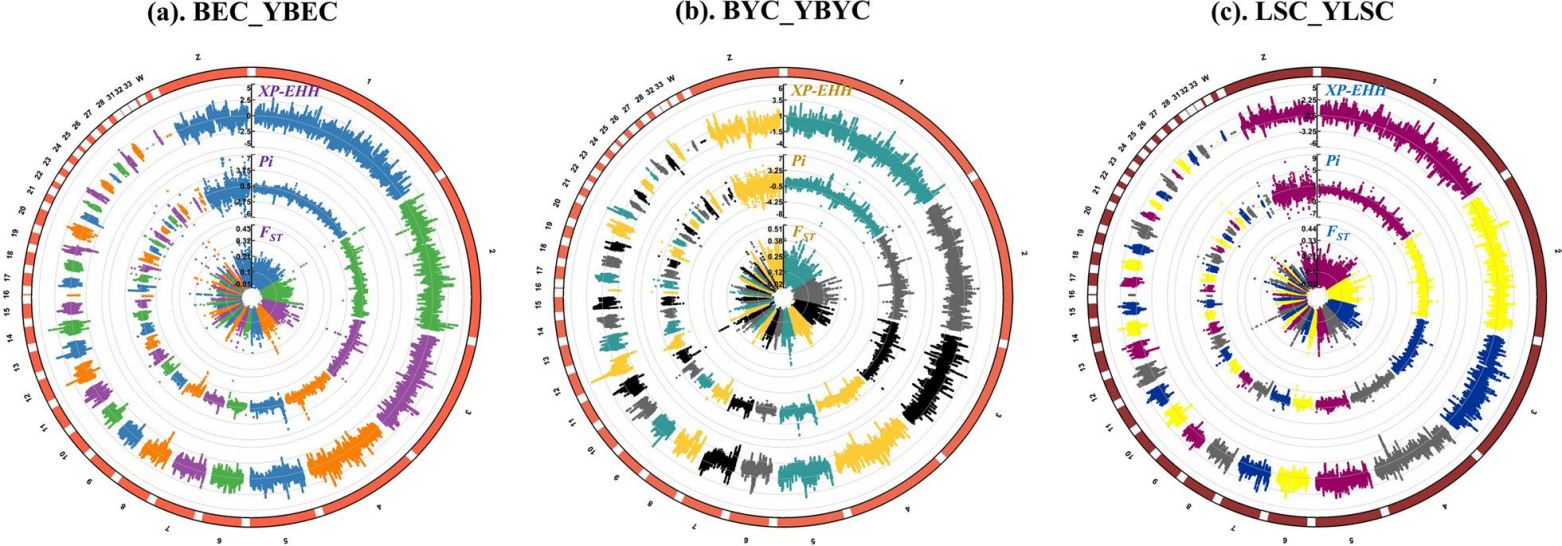
Circos Manhattan plots showing the results for *F*_ST_, Pi, and XP-EHH analyses for the **a** Baier Yellow chicken, **b** Beijing You chicken, and **c** Langshan chicken populations

In order to detect the signals of genetic differentiation, we determined *F*_ST_, *Pi*, and XP-EHH values in 100-kb windows across the genome for the three chicken breeds (Fig. 8). Candidate regions were defined as regions with signals that ranked in the uppermost 5% of values. In order to decrease the number of false positives, only regions identified by all three methods were retained in the final list of positive selection candidates. One hundred and eighty-six, 212, and 161 candidate regions were obtained for the Beijing You, Langshan, and Baier Yellow chicken breeds, respectively (see Additional file 11: Table S6). Genes that may have experienced selection and adaptation were identified in these regions. Protein-coding genes with high *F*_ST_ values (3719 genes), XP-EHH values (4435 genes), and θπ ratios (2504 genes) were identified in the Beijing You chickens, with 857 of these genes identified by all three methods (see Additional file 12: Fig. S6a). Additional file 12: Fig. S6b, c show the corresponding results for the Baier Yellow and Langshan breeds, respectively. Clusterprofiler [63] was used to conduct GO and Kyoto Encyclopedia of Genes and Genomes (KEGG) pathway analyses to investigate potential functions associated with the candidate genes. Significantly enriched GO terms and KEGG pathways are shown in Additional file 13: Fig. S7. In the Beijing You chicken populations, the following enriched GO terms were found: modulation of chemical synaptic transmission (6 genes) and regulation of trans-synaptic signaling (6 genes), and in particular, genes related to the sensory system development, visual system development and eye development were detected. Specific examples are the *RBP4A* and *NOG* genes, for which signatures of selection are supported by all three methods, and which have vital roles in vision and sensory functions. RBP4A is a retinol-binding protein, which is a component of the photopigment in vision cells and is important for maintaining visual function under low light conditions [66, 67]. NOG is a protein coding gene, which plays a role in the lack of neuronal derivatives found in the avian caudal-most neural crest [68]. The KEGG results indicate that the candidate genes are mainly related to the metabolic pathways of amino acids and lipids and in signal transduction pathways. In the Baier Yellow chickens and Langshan chicken populations, significantly enriched GO terms were mainly found for biological pathways such as growth and development, signal transduction, and immune stress response and the KEGG results indicate that the selected genes are mainly enriched in signal pathways such as neurotransmission and amino acid metabolism.

## Discussion

China has 107 distinct domestic breeds of chicken, and because of its long history of animal husbandry and diverse geographical conditions, it has developed extensive genetic resources for this species. Chickens are one of the most widely distributed livestock animals in China. Worldwide, they also have a significant role as a source of income and high-quality protein. The genomes of domestic chickens possess enormous genetic diversity, especially for adaptive traits, including the ability to survive harsh conditions, shifting climate, urbanization, disease epidemics, selection errors, and many other potential stresses [69, 70]. Most Chinese domestic chicken breeds also have unique meat and /or egg qualities, and other useful breed characteristics. However, the majority of these chickens are currently maintained as small populations. Generally, the smaller is a livestock population, the greater is its vulnerability to extinction [5, 71, 72]. Many favorable alleles can be lost as a result of selection or genetic drift. The successful preservation and utilization of these local breeds depend on the accurate assessment of conservation efficiency, which is the essential measure of an effective conservation program. In situ and ex situ approaches to the conservation of animal genetic resources are generally regarded as complementary [73]. Both in situ and ex situ programs have been established for the management of poultry genetic resources in China according to the regulations issued by the Ministry of Agriculture and Rural Affairs. In this study, we used SNPs obtained by high-throughput genome sequencing with an average read depth of 11.28X producing an adequate coverage level to assess the genomic diversity in the chicken populations managed in in situ and ex situ conservation programs, and we provide scientific basis for the optimization of in situ and ex situ conservation programs for domestic chicken breeds in China.

The results show that all three chicken breeds that are part of both in in situ and ex situ conservation programs, have maintained a high level of genetic diversity as measured by heterozygosity (*Ho* and *He*), proportion of polymorphic markers (*P*_*N*_), and allelic richness (*A*_*R*_), which is in agreement with previous studies [7, 34]. Breeds that are conserved in situ show a higher level of genetic diversity than those conserved ex situ. Although conservation time was longer and the in situ populations were larger than the ex situ ones.

Ne is an important measure in genetic conservation practices, and conservation strives to increase it. Numerous methods [74–79] have been developed and applied to estimate Ne across vastly different spatial and temporal scales, ranging from ancient to current population sizes. Here, we estimated Ne based on whole-genome SNPs for the conserved populations and the macrochromosomes (GGA1 to GGA5). For all three breeds, the Ne was larger for chickens enrolled in in situ conservation programs (Fig. 6) and (see Additional file 8: Table S4). We also estimated inbreeding coefficients based on whole-genome SNPs, and found that the inbreeding coefficients for Baier Yellow chickens and Beijing You chickens conserved in situ were lower than for those conserved ex situ, but the opposite was found for the Langshan chickens. This discrepancy may reflect the fact that the duration of the in situ conservation program for the Langshan chicken is the longest among the three breeds, i.e. currently 60 years. The highest inbreeding coefficient, 0.0958, was obtained for the Beijing You chicken (ex situ), which meets our program goals, i.e. maintaining 90% of the whole genome diversity from the initial population, and limiting the inbreeding coefficient to less than 0.1 for 100 years [80].

Based on the NJtree, PCA, STRUCTURE, *F*_ST_ analyses and the distribution of ROH, genetic differentiation has occurred between the two in situ and ex situ conserved populations for all three chicken breeds. The *F*_ST_ values for the three domestic chicken populations ranged from 0.005 to 0.151. There was no genetic differentiation observed over the three consecutive generations of the within-population for any breed, and genetic distance remained relatively close. However, for the three chicken breeds, both the in situ and ex situ conserved populations have reached a moderate degree of differentiation, with *F*_ST_ values ranging from 0.08 to 0.138. In addition, the population structure clustering analysis showed that there was no stratification over the three generations of the ex-situ conserved populations for all three chicken breeds, while obvious genetic stratification was observed for the in situ and ex situ conserved populations of the three breeds. The PCA results showed that the differences were larger between the in situ and ex situ conserved populations of Langshan chickens than for the other two breeds, which may also be related to the fact that the conservation program of the former began the first (in 1959) and has been ongoing for the longest time.

Results from the population structure and fixation index (*F*_ST_) analyses show that all three chicken breeds exhibit genetic differentiation between the in situ and ex situ conserved populations. Since climate and living conditions differ between the populations maintained in in situ and ex situ programs, we hypothesized that genetic adaptation has occurred in response to these changes. Livestock populations that have adapted to different environmental niches (known as ecotypes) cannot always be distinguished easily by their phenotype. Few studies have examined and compared the structures of in situ and ex situ conserved populations, and it is not known how the genetics of domestic chickens may change in response to a shift from the in situ to the ex situ conditions over several decades. To explore the genetic mechanisms underlying the differentiation between the in situ and ex situ conserved chickens, we used *F*_ST_, *Pi* and XP-EHH to detect regions that differed between the two conserved populations. The annotated candidate genes were subjected to KEGG and GO enrichment analysis. The results showed that the Beijing You chicken population, was enriched in the GO terms of the sensory system development, visual system development, and eye development, which may be due to adaptation of this population to changes in environmental factors such as temperature and humidity after migration from its original location to the site of the gene bank. The candidate genes that were detected from the analyses of the in situ and ex situ conserved populations of Baier Yellow chicken and Langshan chicken were mainly enriched in regulatory pathways related to energy metabolism, signal transduction, and immunity. Based on the functions of the genes revealed by the KEGG and GO term analyses, we hypothesize that the genetic differences may be related to adaptation to local environmental conditions. For example, the conservation of the Beijing You chicken began at the BAAFS institute of Animal Husbandry and Veterinary Medicine in 1972. This in situ conservation program reached its 47th year in 2018. In 1976, Beijing You chickens were obtained from the Beijing program and transferred to Yangzhou, Jiangsu (National Chickens Genetic Resources) to establish an ex situ conservation program, which reached its 40th year in 2015. Climate conditions (such as light, temperature, and humidity) in the two locations are markedly different. In contrast, the conservation programs for the Baier Yellow chickens and Langshan chickens were conducted under nearly identical climate conditions at Zhejiang and Jiangsu. The population sizes for these chickens were very small at the onset of the conservation program, so that the genetic differentiation may have been caused by genetic drift over several decades. Alternatively, given the very small founder populations used in these programs, different variants may have been sampled from the original populations simply by chance.

Conserving the biodiversity of native poultry breeds is becoming a matter of great concern worldwide. The Food and Agriculture Organization (FAO) of the United Nations has drawn attention to the alarming trend of local livestock breeds disappearing in the world and has estimated that 40 breeds of chicken have become extinct [81, 82]. Over the last decades, only 15% of the countries have poultry conservation programs, which cover 63% of local breeds and 11% of national populations of transboundary breeds [83]. In a study using a 57 K SNP chip, Restoux et al. [84] demonstrated that both the between- and within-breed genetic diversity levels are high in the French local chicken populations which is consistent with our findings. However, in some developing countries, monitoring the efficiency of conservation programs has been based on conservation parameters, but the full potential of the genetic and molecular techniques has to be considered in view of the limited available budgets [85]. To sum up, our study not only provides a valuable reference for evaluating the current conservation chicken programs in China, but also proves that these are more effective than in other countries.

## Conclusions

Maintaining the genomic diversity of Chinese domestic chicken breeds is important for economic and cultural reasons. In this study, we conducted genotyping-by-sequencing analysis for three Chinese domestic chicken breeds that are conserved in in situ and ex situ conditions with different conservation programs. We found that these current conservation programs have maintained the genomic diversity of these three Chinese domestic chicken breeds. The small ex situ conserved populations that are maintained in controlled environments retain less genetic diversity than populations conserved in in situ*.* In addition, the transfer of conservation populations from their place of origin to another site results in genetic differentiation, which may be caused by genetic drift or adaptation. The results of this study provide a basis for further optimization of conservation programs for domestic chicken breeds.

### Supplementary Information

**Additional file 1: Table S1.** Summary statistics for genome sequencing.

**Additional file 2: Figure S1**. **a** SNP density and distribution across the genome; and **b** Number of novel SNPs vs. those found within the dbSNP database.

**Additional file 3: Figure S2.** Indel density and distribution across the genome.

**Additional file 4: Table S2.** Summary of genome sequencing and annotation of variants for the three Chinese domestic chicken breeds.

**Additional file 5: Figure S3.** Biplots showing PC1 vs. PC2, PC1 vs PC3, and PC2 vs PC3.

**Additional file 6: Figure S4.** The CV error associated with each K value.

**Additional file 7: Table S3.** Estimation of the pairwise genetic differentiation statistic among breeds (*F*_ST_).

**Additional file 8: Table S4.** Effective population size (Ne) estimated for the three breeds in in situ and ex situ conservation programs.

**Additional file 9: Table S5.** Statistical summary of analysis for runs of homozygosity in in situ and ex situ conserved chicken populations.

**Additional file 10: Figure S5.** Circos plot showing genomic location of runs of homozygosity for each of the three chicken breeds in in situ and ex situ conserved populations.

**Additional file 11: Table S6.** Candidate regions in **a** Baier Yellow chicken; **b** Beijing You chicken; and **c** Langshan chicken.

**Additional file 12: Figure S6.** Venn diagrams showing numbers of genes identified using *F*_ST_, Pi, and XP-EHH analyses for **a** Baier Yellow chicken: **b** Beijing You chicken; and (c) Langshan chicken.

**Additional file 13: Figure S7**. Go term and KEGG analysis for **a** Beijing You chicken; **b** Baier Yellow chicken; and **c** Langshan chicken.

## Data Availability

The datasets used for the current study are available from the corresponding author upon reasonable request.
