# Supplementary material for: Managing genomic diversity in conservation programs of Chinese domestic chickens
Source: Genet Sel Evol. 2023 Dec 14;55:92. doi: 10.1186/s12711-023-00866-3 (PMC10722821; doi:10.1186/s12711-023-00866-3)
Supplement: Supplementary file 1 — Additional file 1: Table S1. Summary statistics for genome sequencing. [file 12711_2023_866_MOESM1_ESM.doc]

**Additional file 1: Table S1 Data statistics of genome sequencing**

| Group | Sample | Total_Reads | Mapped_Reads | Mapping_Rate(%) | Raw Base(bp) | Clean Base(bp) | Effective Rate(%) | Average_Depth | Coverage_at_least 1X | Coverage_at_least_4X | Q20 | Q30 | GC Content (%) |
| --- | --- | --- | --- | --- | --- | --- | --- | --- | --- | --- | --- | --- | --- |
| YBYC | YBYC1 | 5,492,192 | 5,463,429 | 99.48 | 790,935,264 | 790,875,648 | 99.99 | 7.03 | 9.13 | 5.46 | 95.52 | 88.85 | 39.93 |
| YBYC | YBYC2 | 4,638,754 | 4,611,528 | 99.41 | 668,033,568 | 667,980,576 | 99.99 | 5.43 | 9.96 | 5.11 | 95 | 87.66 | 40.15 |
| YBYC | YBYC3 | 4,804,434 | 4,788,666 | 99.67 | 691,884,288 | 691,838,496 | 99.99 | 5.79 | 9.72 | 5.09 | 93.96 | 85 | 40.64 |
| YBYC | YBYC4 | 5,563,292 | 5,535,471 | 99.50 | 801,168,768 | 801,114,048 | 99.99 | 6.06 | 10.72 | 5.65 | 95.71 | 89.27 | 40.25 |
| YBYC | YBYC5 | 5,609,520 | 5,583,532 | 99.54 | 807,827,904 | 807,770,880 | 99.99 | 6.52 | 10.06 | 5.60 | 95.77 | 89.35 | 40.27 |
| YBYC | YBYC6 | 4,869,512 | 4,849,666 | 99.59 | 701,209,728 | 701,209,728 | 100 | 5.25 | 10.75 | 5.01 | 95.39 | 88.21 | 40.41 |
| YBYC | YBYC7 | 6,894,332 | 6,852,393 | 99.39 | 994,198,464 | 992,783,808 | 99.86 | 7.89 | 10.20 | 5.96 | 95.55 | 88.83 | 40.24 |
| YBYC | YBYC8 | 5,520,572 | 5,501,498 | 99.65 | 794,962,944 | 794,962,368 | 100 | 6.00 | 10.73 | 5.41 | 97.02 | 91.89 | 40.42 |
| YBYC | YBYC9 | 5,056,940 | 5,040,783 | 99.68 | 728,199,360 | 728,199,360 | 100 | 6.46 | 9.18 | 5.18 | 97.2 | 92.31 | 40.53 |
| YBYC | YBYC10 | 6,582,788 | 6,559,941 | 99.65 | 947,921,472 | 947,921,472 | 100 | 7.73 | 9.97 | 5.85 | 96.32 | 90.29 | 40.52 |
| YBYC | YBYC11 | 7,069,384 | 7,024,699 | 99.37 | 1,019,360,448 | 1,017,991,296 | 99.87 | 8.25 | 9.99 | 5.98 | 94.19 | 85.88 | 39.95 |
| YBYC | YBYC12 | 7,299,202 | 7,244,458 | 99.25 | 1,052,523,936 | 1,051,085,088 | 99.86 | 8.42 | 10.10 | 6.03 | 94.34 | 86.36 | 40.2 |
| YBYC | YBYC13 | 7,291,092 | 7,263,275 | 99.62 | 1,049,917,536 | 1,049,917,248 | 100 | 7.31 | 11.67 | 6.49 | 96.38 | 90.5 | 40.23 |
| YBYC | YBYC14 | 7,293,992 | 7,267,099 | 99.63 | 1,050,334,848 | 1,050,334,848 | 100 | 7.99 | 10.68 | 6.16 | 95.8 | 89.14 | 40.52 |
| YBYC | YBYC15 | 8,876,476 | 8,842,179 | 99.61 | 1,280,014,272 | 1,278,212,544 | 99.86 | 9.43 | 11.01 | 6.47 | 93.88 | 85 | 40.45 |
| YBYC | YBYC16 | 5,904,698 | 5,880,512 | 99.59 | 850,276,512 | 850,276,512 | 100 | 6.57 | 10.51 | 5.63 | 95.42 | 88.26 | 40.78 |
| YBYC | YBYC17 | 4,501,372 | 4,494,339 | 99.84 | 649,183,680 | 648,197,568 | 99.85 | 6.19 | 8.54 | 4.98 | 94.08 | 85 | 39.91 |
| YBYC | YBYC18 | 7,345,062 | 7,319,554 | 99.65 | 1,057,688,928 | 1,057,688,928 | 100 | 8.23 | 10.45 | 6.06 | 96.35 | 90.32 | 40.2 |
| YBYC | YBYC19 | 6,902,964 | 6,875,619 | 99.60 | 994,027,104 | 994,026,816 | 100 | 7.85 | 10.29 | 5.96 | 95.66 | 88.8 | 40.53 |
| YBYC | YBYC20 | 6,751,174 | 6,727,279 | 99.65 | 972,169,056 | 972,169,056 | 100 | 7.80 | 10.13 | 5.82 | 94.4 | 85.87 | 40.75 |
| YBYC | YBYC21 | 5,398,594 | 5,378,842 | 99.63 | 777,397,536 | 777,397,536 | 100 | 5.96 | 10.59 | 5.43 | 96.56 | 90.82 | 40.75 |
| YBYC | YBYC22 | 10,022,254 | 9,953,584 | 99.31 | 1,445,005,728 | 1,443,204,576 | 99.88 | 10.32 | 11.32 | 6.76 | 94.35 | 86.31 | 40.2 |
| YBYC | YBYC23 | 6,609,802 | 6,588,404 | 99.68 | 951,811,488 | 951,811,488 | 100 | 7.58 | 10.22 | 5.85 | 96.54 | 90.78 | 40.66 |
| YBYC | YBYC24 | 4,428,106 | 4,410,511 | 99.60 | 637,647,264 | 637,647,264 | 100 | 6.30 | 8.22 | 4.56 | 96.3 | 90.27 | 40.17 |
| YBYC | YBYC25 | 4,043,514 | 4,028,944 | 99.64 | 582,266,304 | 582,266,016 | 100 | 5.29 | 8.94 | 4.55 | 96.39 | 90.4 | 40.5 |
| YBYC | YBYC26 | 6,273,100 | 6,247,241 | 99.59 | 903,326,400 | 903,326,400 | 100 | 7.01 | 10.47 | 5.81 | 96.09 | 89.83 | 40.67 |
| YBYC | YBYC27 | 5,935,412 | 5,914,865 | 99.65 | 854,699,328 | 854,699,328 | 100 | 7.19 | 9.66 | 5.39 | 96.66 | 90.99 | 40.51 |
| YBYC | YBYC28 | 5,697,210 | 5,677,184 | 99.65 | 820,398,528 | 820,398,240 | 100 | 6.69 | 9.96 | 5.45 | 96.6 | 90.88 | 40.64 |
| YBYC | YBYC29 | 4,901,924 | 4,883,681 | 99.63 | 705,877,632 | 705,877,056 | 100 | 5.51 | 10.40 | 5.16 | 96.39 | 90.44 | 40.78 |
| YBYC | YBYC30 | 5,063,350 | 5,044,977 | 99.64 | 729,122,400 | 729,122,400 | 100 | 5.56 | 10.66 | 5.26 | 95.81 | 89.08 | 40.67 |
|  |  |  |  |  |  |  |  |  |  |  |  |  |  |
| YBEC | YBEC1 | 4,000,234 | 3,980,509 | 99.51 | 576,078,624 | 576,033,696 | 99.99 | 4.86 | 9.62 | 4.66 | 95.62 | 89.06 | 40.23 |
| YBEC | YBEC2 | 4,056,378 | 4,036,292 | 99.50 | 584,154,144 | 584,118,432 | 99.99 | 5.05 | 9.37 | 4.68 | 95.04 | 87.65 | 40.23 |
| YBEC | YBEC3 | 4,365,410 | 4,348,878 | 99.62 | 628,665,984 | 628,619,040 | 99.99 | 5.53 | 9.24 | 4.91 | 96.41 | 90.75 | 40.02 |
| YBEC | YBEC4 | 4,671,560 | 4,650,992 | 99.56 | 672,755,040 | 672,704,640 | 99.99 | 6.04 | 9.05 | 5.06 | 95.75 | 89.31 | 40.27 |
| YBEC | YBEC5 | 4,934,268 | 4,912,448 | 99.56 | 710,587,872 | 710,534,592 | 99.99 | 6.08 | 9.49 | 5.24 | 96 | 89.91 | 40.13 |
| YBEC | YBEC6 | 3,704,584 | 3,688,066 | 99.55 | 533,500,416 | 533,460,096 | 99.99 | 4.69 | 9.23 | 4.42 | 95.92 | 89.67 | 40.22 |
| YBEC | YBEC7 | 4,662,696 | 4,641,892 | 99.55 | 671,479,776 | 671,428,224 | 99.99 | 5.90 | 9.23 | 4.96 | 95.59 | 88.84 | 39.84 |
| YBEC | YBEC8 | 4,412,628 | 4,390,137 | 99.49 | 635,462,496 | 635,418,432 | 99.99 | 5.28 | 9.74 | 4.85 | 95.21 | 88.06 | 39.98 |
| YBEC | YBEC9 | 4,272,694 | 4,248,982 | 99.45 | 615,318,912 | 615,267,936 | 99.99 | 5.12 | 9.75 | 4.87 | 95.14 | 87.84 | 40.3 |
| YBEC | YBEC10 | 4,048,006 | 4,027,927 | 99.50 | 582,950,880 | 582,912,864 | 99.99 | 5.09 | 9.29 | 4.65 | 95.09 | 87.77 | 40.38 |
| YBEC | YBEC11 | 4,303,326 | 4,281,989 | 99.50 | 619,719,840 | 619,678,944 | 99.99 | 5.42 | 9.28 | 4.77 | 95.56 | 88.89 | 40.51 |
| YBEC | YBEC12 | 4,453,718 | 4,430,985 | 99.49 | 641,378,880 | 641,335,392 | 99.99 | 5.39 | 9.65 | 4.92 | 96.06 | 90.09 | 40.17 |
| YBEC | YBEC13 | 4,731,184 | 4,706,750 | 99.48 | 681,338,880 | 681,290,496 | 99.99 | 5.96 | 9.28 | 5.06 | 95.69 | 89.24 | 40.17 |
| YBEC | YBEC14 | 10,179,760 | 10,107,289 | 99.29 | 1,467,907,488 | 1,465,885,440 | 99.86 | 9.93 | 11.94 | 6.96 | 94.13 | 85.91 | 40.36 |
| YBEC | YBEC15 | 4,223,322 | 4,199,787 | 99.44 | 608,202,432 | 608,158,368 | 99.99 | 5.60 | 8.81 | 4.74 | 95.59 | 89.09 | 40.11 |
| YBEC | YBEC16 | 6,958,730 | 6,947,527 | 99.84 | 1,003,502,304 | 1,002,057,120 | 99.86 | 7.53 | 10.84 | 6.01 | 94.05 | 85 | 40.53 |
| YBEC | YBEC17 | 4,570,140 | 4,550,661 | 99.57 | 658,152,864 | 658,100,160 | 99.99 | 5.36 | 9.96 | 5.03 | 96.31 | 90.61 | 40.13 |
| YBEC | YBEC18 | 5,197,776 | 5,171,738 | 99.50 | 748,535,904 | 748,479,744 | 99.99 | 6.14 | 9.86 | 5.30 | 95.89 | 89.69 | 40.13 |
| YBEC | YBEC19 | 4,633,110 | 4,604,652 | 99.39 | 667,212,480 | 667,167,840 | 99.99 | 5.65 | 9.57 | 5.01 | 94.78 | 87.22 | 40.26 |
| YBEC | YBEC20 | 3,395,454 | 3,376,739 | 99.45 | 488,987,136 | 488,945,376 | 99.99 | 4.57 | 8.68 | 4.08 | 95.15 | 88 | 40.38 |
| YBEC | YBEC21 | 4,793,548 | 4,775,989 | 99.63 | 690,270,912 | 690,270,912 | 100 | 5.15 | 10.88 | 5.11 | 95.88 | 88.78 | 41.07 |
| YBEC | YBEC22 | 4,895,040 | 4,870,235 | 99.49 | 704,935,008 | 704,885,760 | 99.99 | 5.79 | 9.87 | 5.20 | 95.55 | 88.89 | 40.31 |
| YBEC | YBEC23 | 3,976,612 | 3,953,020 | 99.41 | 572,673,312 | 572,632,128 | 99.99 | 4.90 | 9.47 | 4.58 | 94.63 | 86.87 | 40.37 |
| YBEC | YBEC24 | 4,890,832 | 4,869,952 | 99.57 | 704,330,496 | 704,279,808 | 99.99 | 6.02 | 9.50 | 5.16 | 96.22 | 90.41 | 40.32 |
| YBEC | YBEC25 | 4,180,334 | 4,162,708 | 99.58 | 602,011,008 | 601,968,096 | 99.99 | 5.36 | 9.13 | 4.74 | 96.41 | 90.85 | 40.17 |
| YBEC | YBEC26 | 4,661,958 | 4,640,709 | 99.54 | 671,369,184 | 671,321,952 | 99.99 | 5.62 | 9.71 | 5.05 | 95.58 | 88.86 | 40.34 |
| YBEC | YBEC27 | 5,880,524 | 5,843,768 | 99.37 | 847,924,416 | 846,795,456 | 99.87 | 6.99 | 9.82 | 5.59 | 95.2 | 88.15 | 40.28 |
| YBEC | YBEC28 | 4,822,082 | 4,797,431 | 99.49 | 694,431,072 | 694,379,808 | 99.99 | 5.60 | 10.06 | 5.18 | 95.7 | 89.27 | 40.26 |
| YBEC | YBEC29 | 4,501,922 | 4,477,992 | 99.47 | 648,323,424 | 648,276,768 | 99.99 | 5.66 | 9.29 | 4.91 | 95.06 | 87.77 | 40.36 |
| YBEC | YBEC30 | 3,965,884 | 3,941,841 | 99.39 | 571,123,296 | 571,087,296 | 99.99 | 4.78 | 9.69 | 4.55 | 94.62 | 86.78 | 40.47 |
|  |  |  |  |  |  |  |  |  |  |  |  |  |  |
| YLSC | YLSC1 | 5,632,288 | 5,610,808 | 99.62 | 811,049,760 | 811,049,472 | 100 | 5.30 | 12.41 | 5.90 | 94.88 | 86.42 | 41.01 |
| YLSC | YLSC2 | 5,922,318 | 5,900,393 | 99.63 | 852,813,792 | 852,813,792 | 100 | 5.49 | 12.62 | 6.08 | 95.42 | 87.7 | 41.14 |
| YLSC | YLSC3 | 5,839,272 | 5,798,566 | 99.30 | 841,961,664 | 840,855,168 | 99.87 | 7.50 | 9.06 | 5.38 | 94.25 | 86.1 | 39.63 |
| YLSC | YLSC4 | 5,563,292 | 5,535,471 | 99.50 | 1,085,472,288 | 1,084,000,896 | 99.86 | 6.06 | 10.72 | 5.65 | 94.27 | 86.07 | 40.25 |
| YLSC | YLSC5 | 5,609,520 | 5,583,532 | 99.54 | 1,203,030,720 | 1,201,351,392 | 99.86 | 6.52 | 10.06 | 5.60 | 93.88 | 85.39 | 40.45 |
| YLSC | YLSC6 | 4,869,512 | 4,849,666 | 99.59 | 1,252,814,976 | 1,251,182,016 | 99.87 | 5.25 | 10.75 | 5.01 | 94.69 | 86.97 | 39.93 |
| YLSC | YLSC7 | 9,154,680 | 9,093,587 | 99.33 | 1,320,014,880 | 1,318,273,920 | 99.87 | 10.04 | 10.62 | 6.49 | 94.62 | 86.85 | 40.14 |
| YLSC | YLSC8 | 8,380,660 | 8,323,552 | 99.32 | 1,208,453,760 | 1,206,815,040 | 99.86 | 9.04 | 10.81 | 6.31 | 94.32 | 86.2 | 40.41 |
| YLSC | YLSC9 | 8,260,708 | 8,236,729 | 99.71 | 1,191,247,488 | 1,189,541,952 | 99.86 | 8.99 | 10.76 | 6.29 | 93.97 | 85 | 40.62 |
| YLSC | YLSC10 | 8,479,134 | 8,432,623 | 99.45 | 1,222,588,224 | 1,220,995,296 | 99.87 | 8.80 | 11.25 | 6.45 | 95.44 | 88.49 | 40.5 |
| YLSC | YLSC11 | 8,934,438 | 8,878,121 | 99.37 | 1,288,270,080 | 1,286,559,072 | 99.87 | 9.14 | 11.40 | 6.55 | 94.59 | 86.78 | 40.51 |
| YLSC | YLSC12 | 9,177,896 | 9,119,616 | 99.36 | 1,323,423,648 | 1,321,617,024 | 99.86 | 9.37 | 11.43 | 6.57 | 94.96 | 87.58 | 40.6 |
| YLSC | YLSC13 | 9,189,578 | 9,134,991 | 99.41 | 1,325,109,600 | 1,323,299,232 | 99.86 | 9.24 | 11.60 | 6.63 | 94.77 | 87.09 | 40.55 |
| YLSC | YLSC14 | 8,131,080 | 8,081,423 | 99.39 | 1,172,368,224 | 1,170,875,520 | 99.87 | 8.82 | 10.74 | 6.18 | 94.25 | 85.88 | 40.09 |
| YLSC | YLSC15 | 2,200,722 | 2,188,773 | 99.46 | 316,931,040 | 316,903,968 | 99.99 | 3.66 | 7.02 | 2.74 | 95.53 | 88.85 | 39.78 |
| YLSC | YLSC16 | 6,117,970 | 6,088,245 | 99.51 | 882,184,608 | 880,987,680 | 99.86 | 7.41 | 9.64 | 5.64 | 93.81 | 85 | 40.16 |
| YLSC | YLSC17 | 3,600,386 | 3,582,735 | 99.51 | 518,494,176 | 518,455,584 | 99.99 | 4.85 | 8.66 | 4.27 | 95.58 | 88.94 | 40.28 |
| YLSC | YLSC18 | 8,564,618 | 8,526,225 | 99.55 | 1,234,958,976 | 1,233,304,992 | 99.87 | 8.76 | 11.42 | 6.50 | 93.92 | 85 | 40.6 |
| YLSC | YLSC19 | 8,169,946 | 8,141,136 | 99.65 | 1,178,124,768 | 1,176,472,224 | 99.86 | 8.53 | 11.21 | 6.38 | 93.91 | 85 | 40.63 |
| YLSC | YLSC20 | 9,219,268 | 9,157,588 | 99.33 | 1,329,392,736 | 1,327,574,592 | 99.86 | 9.45 | 11.37 | 6.60 | 94.29 | 86.17 | 40.57 |
| YLSC | YLSC21 | 10,040,658 | 9,968,415 | 99.28 | 1,447,883,424 | 1,445,854,752 | 99.86 | 9.79 | 11.95 | 6.85 | 95 | 87.77 | 40.42 |
| YLSC | YLSC22 | 9,440,638 | 9,372,116 | 99.27 | 1,361,263,968 | 1,359,451,872 | 99.87 | 9.75 | 11.28 | 6.63 | 94.45 | 86.56 | 40.44 |
| YLSC | YLSC23 | 4,283,348 | 4,259,070 | 99.43 | 616,843,872 | 616,802,112 | 99.99 | 5.57 | 8.98 | 4.77 | 95.29 | 88.32 | 40.32 |
| YLSC | YLSC24 | 4,376,786 | 4,356,431 | 99.53 | 630,309,024 | 630,257,184 | 99.99 | 5.54 | 9.23 | 4.79 | 95.83 | 89.48 | 40.03 |
| YLSC | YLSC25 | 12,690,618 | 12,591,221 | 99.22 | 1,830,019,968 | 1,827,448,992 | 99.86 | 11.33 | 13.03 | 7.42 | 94.34 | 86.36 | 40.27 |
| YLSC | YLSC26 | 8,671,482 | 8,616,252 | 99.36 | 1,250,378,208 | 1,248,693,408 | 99.87 | 8.84 | 11.43 | 6.53 | 95.3 | 88.39 | 40.49 |
| YLSC | YLSC27 | 9,560,084 | 9,495,236 | 99.32 | 1,378,565,280 | 1,376,652,096 | 99.86 | 8.83 | 12.62 | 6.92 | 94.72 | 87.12 | 40.49 |
| YLSC | YLSC28 | 8,603,016 | 8,583,874 | 99.78 | 1,240,646,976 | 1,238,834,304 | 99.85 | 8.90 | 11.33 | 6.56 | 94 | 85 | 40.37 |
| YLSC | YLSC29 | 6,600,838 | 6,581,010 | 99.70 | 507,435,552 | 507,393,504 | 99.99 | 7.30 | 10.59 | 5.93 | 95.81 | 89.47 | 39.98 |
| YLSC | YLSC30 | 3,523,566 | 3,506,584 | 99.52 | 1,316,229,984 | 1,314,396,576 | 99.86 | 5.01 | 8.22 | 4.27 | 93.76 | 85 | 40.41 |
| YLSC | YLSC31 | 9,127,754 | 9,079,582 | 99.47 | 811,049,760 | 811,049,472 | 100 | 8.99 | 11.85 | 6.68 | 94.88 | 86.42 | 41.01 |
