# Supplementary figures and images for: Managing genomic diversity in conservation programs of Chinese domestic chickens
Source: Genet Sel Evol. 2023 Dec 14;55:92. doi: 10.1186/s12711-023-00866-3 (PMC10722821; doi:10.1186/s12711-023-00866-3)

**(a)**

# The number of SNPs within 0.1Mb window size

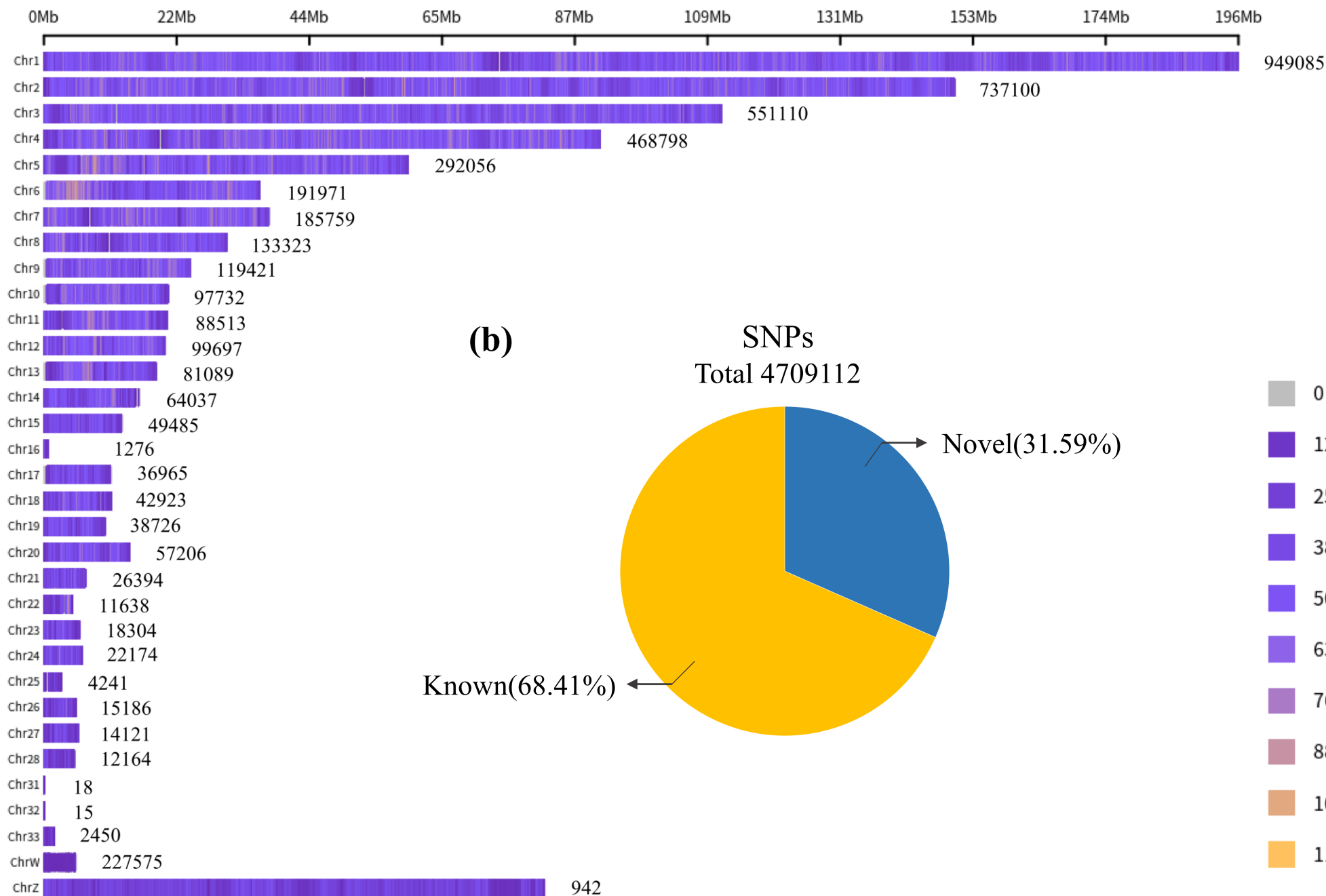**(b)**

## SNPs Total 4709112

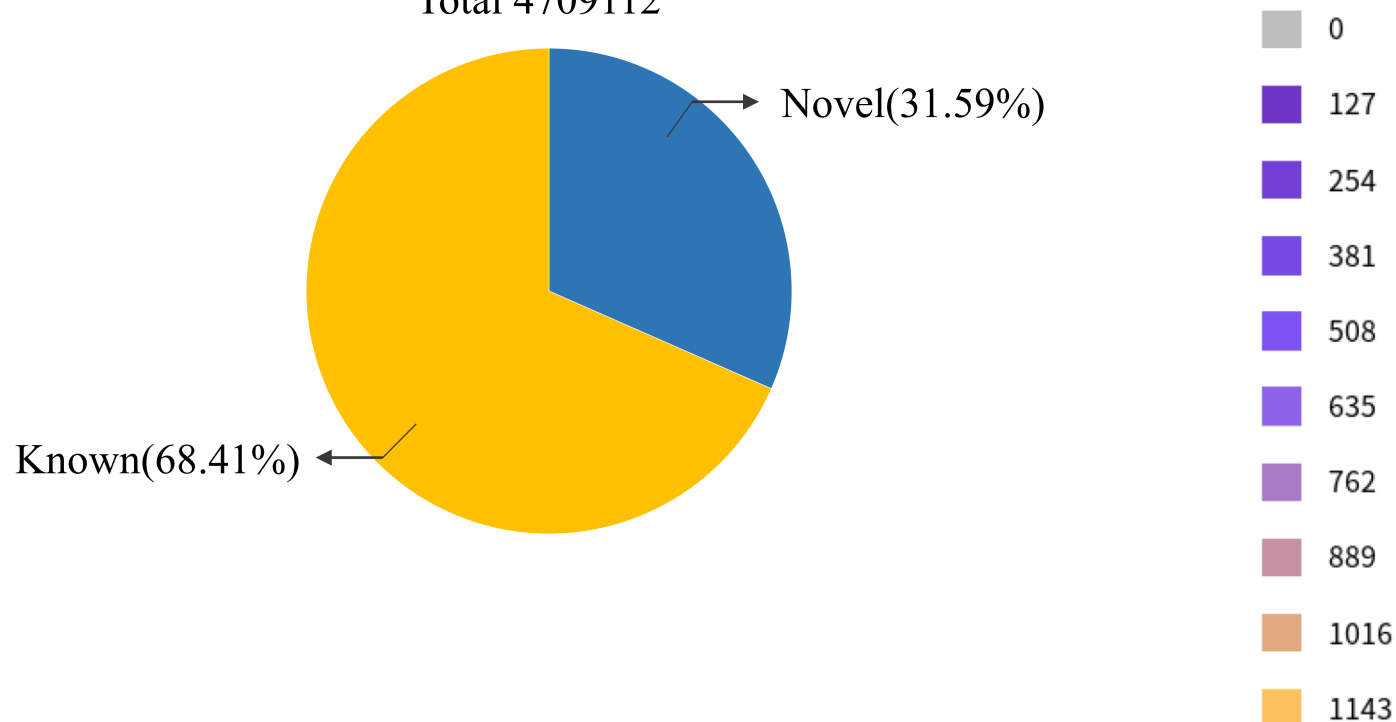

Supplement: Supplementary file 2 — Additional file 2: Figure S1. a SNP density and distribution across the genome; and b Number of novel SNPs vs. those found within the dbSNP database. [file 12711_2023_866_MOESM2_ESM.pdf]

# The number of Indels within 0.1Mb window size

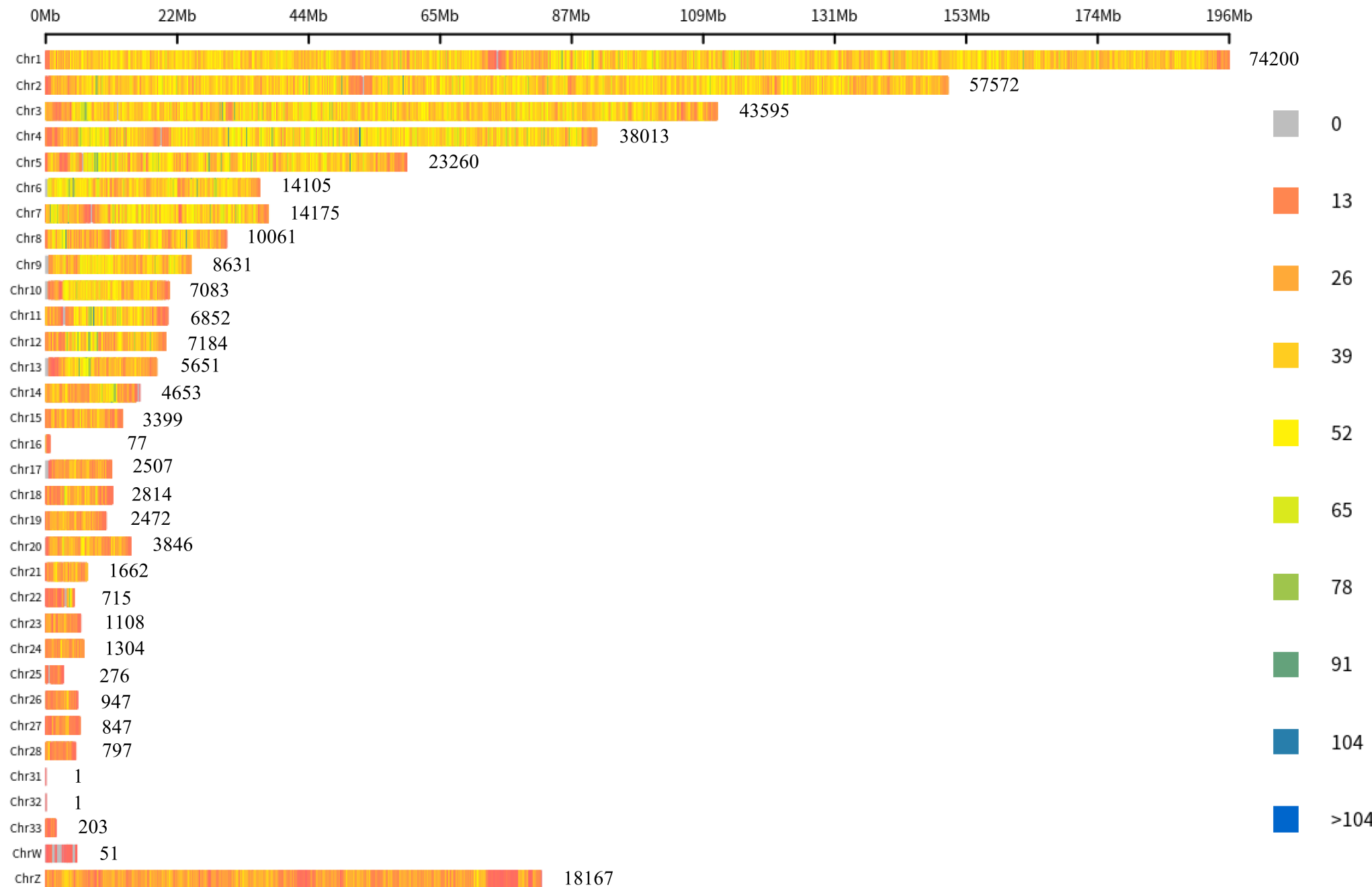

Supplement: Supplementary file 3 — Additional file 3: Figure S2. Indel density and distribution across the genome. [file 12711_2023_866_MOESM3_ESM.pdf]

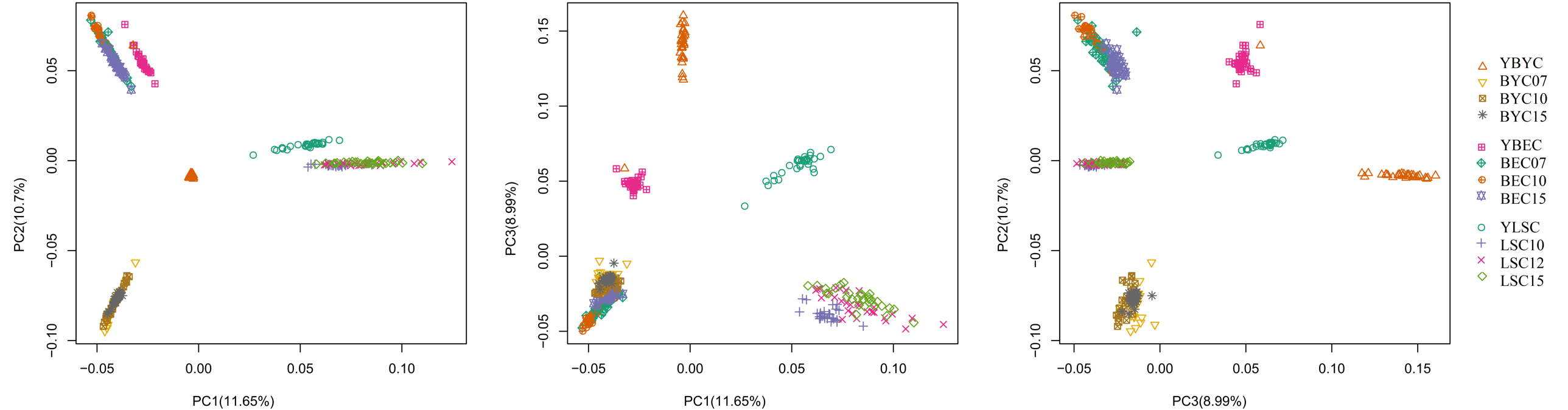

Supplement: Supplementary file 5 — Additional file 5: Figure S3. Biplots showing PC1 vs. PC2, PC1 vs PC3, and PC2 vs PC3. [file 12711_2023_866_MOESM5_ESM.pdf]

## CV error

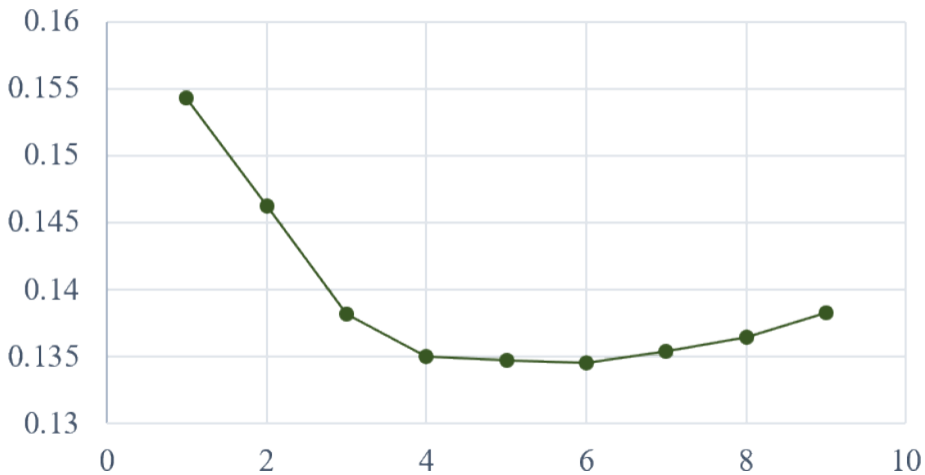

Supplement: Supplementary file 6 — Additional file 6: Figure S4. The CV error associated with each K value. [file 12711_2023_866_MOESM6_ESM.pdf]

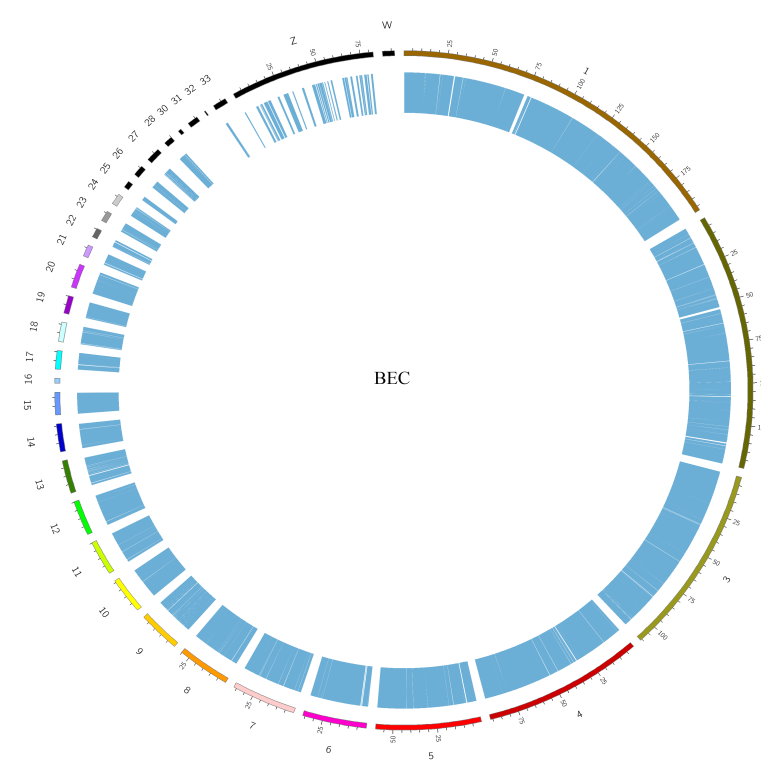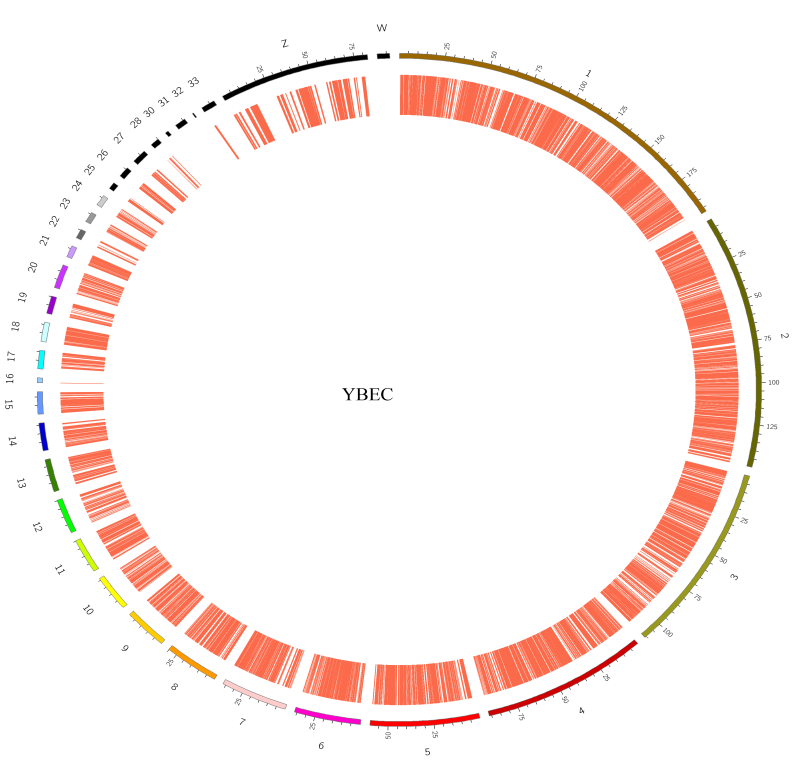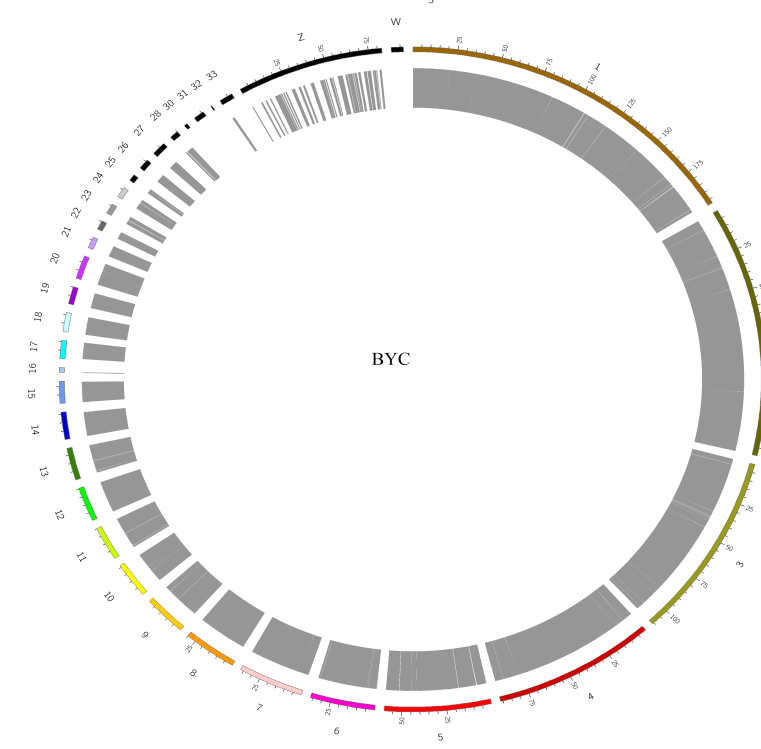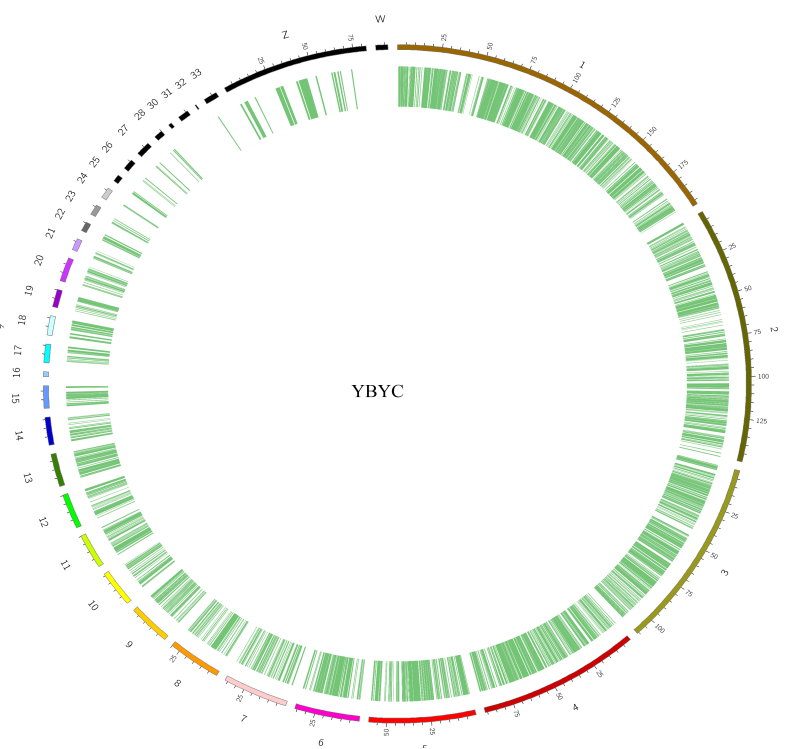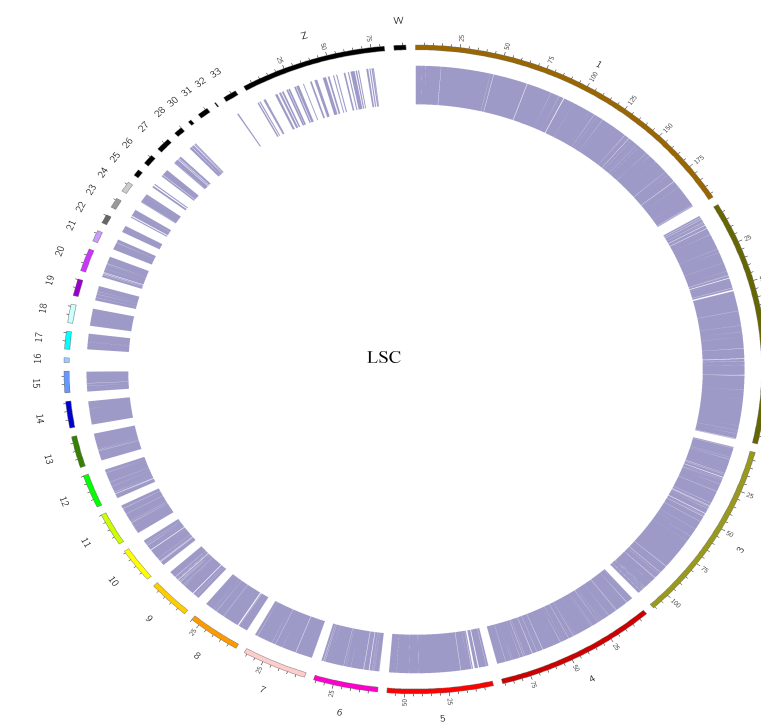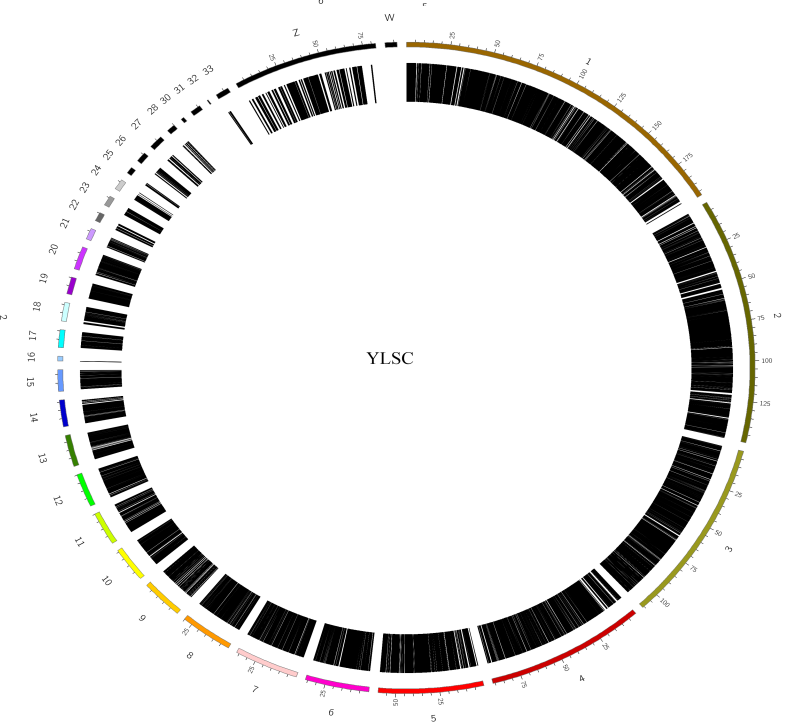

Supplement: Supplementary file 10 — Additional file 10: Figure S5. Circos plot showing genomic location of runs of homozygosity for each of the three chicken breeds in in situ and ex situ conserved populations. [file 12711_2023_866_MOESM10_ESM.pdf]

**(a). BEC\_YBEC**

**FST**

**Pi**

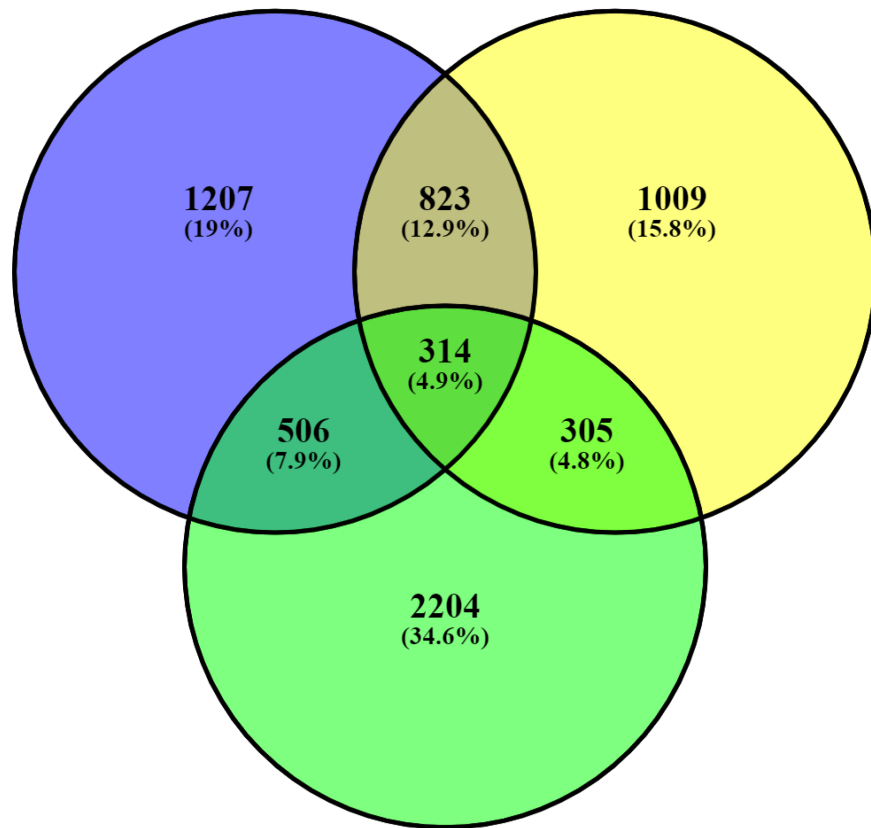

**XP-EHH**

**(b). BYC\_YBYC**

**FST**

**Pi**

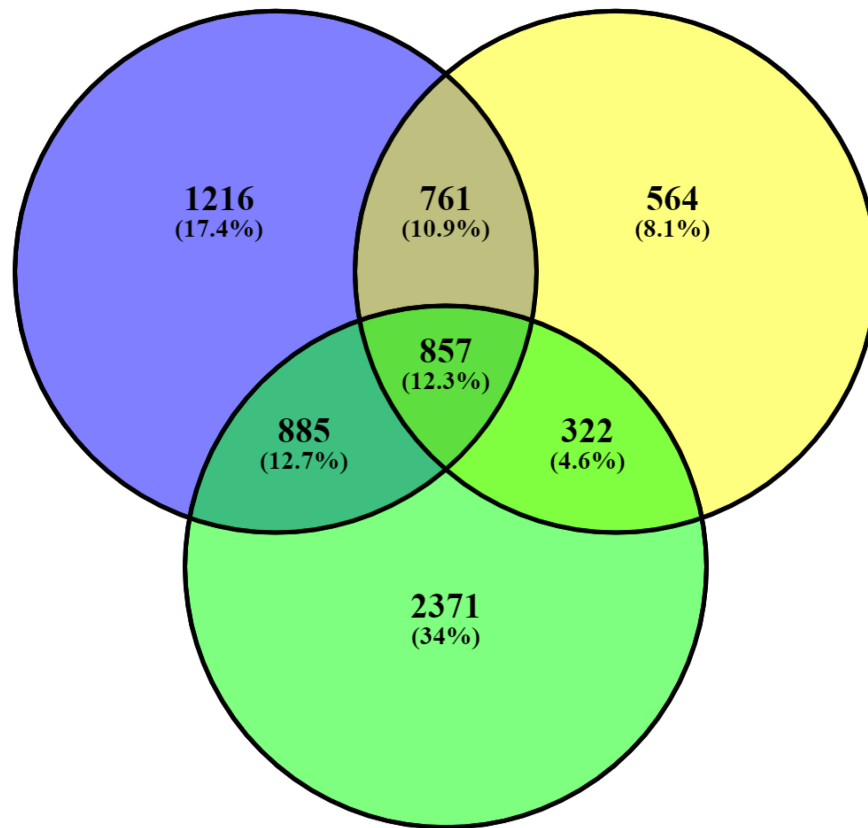

**XP-EHH**

**(c). LSC\_YLSC**

**FST**

**Pi**

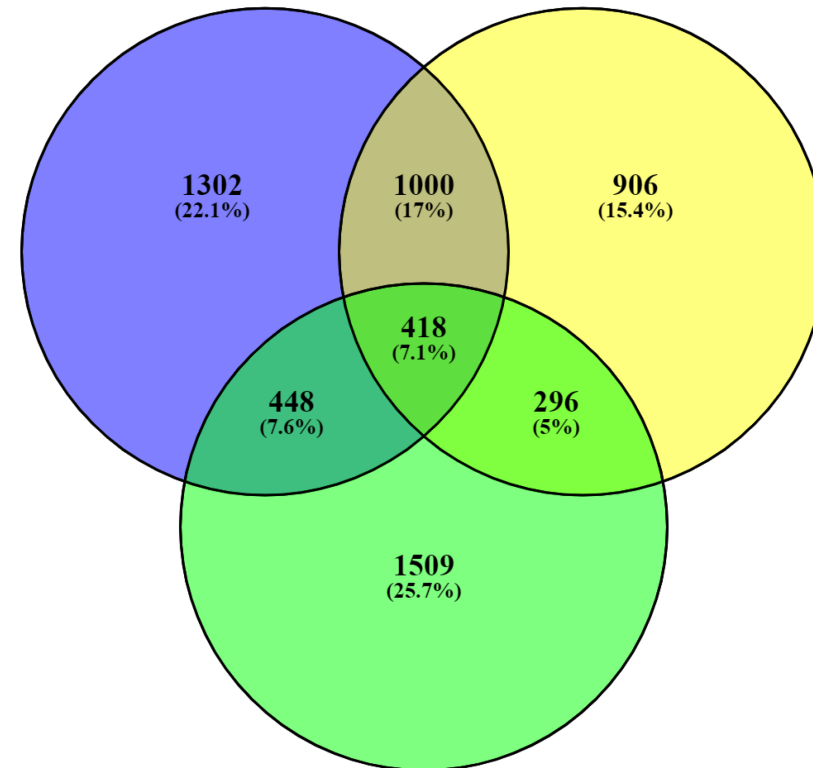

**XP-EHH**

Supplement: Supplementary file 12 — Additional file 12: Figure S6. Venn diagrams showing numbers of genes identified using FST, Pi, and XP-EHH analyses for a Baier Yellow chicken: b Beijing You chicken; and (c) Langshan chicken. [file 12711_2023_866_MOESM12_ESM.pdf]

(a). Beijing You Chicken

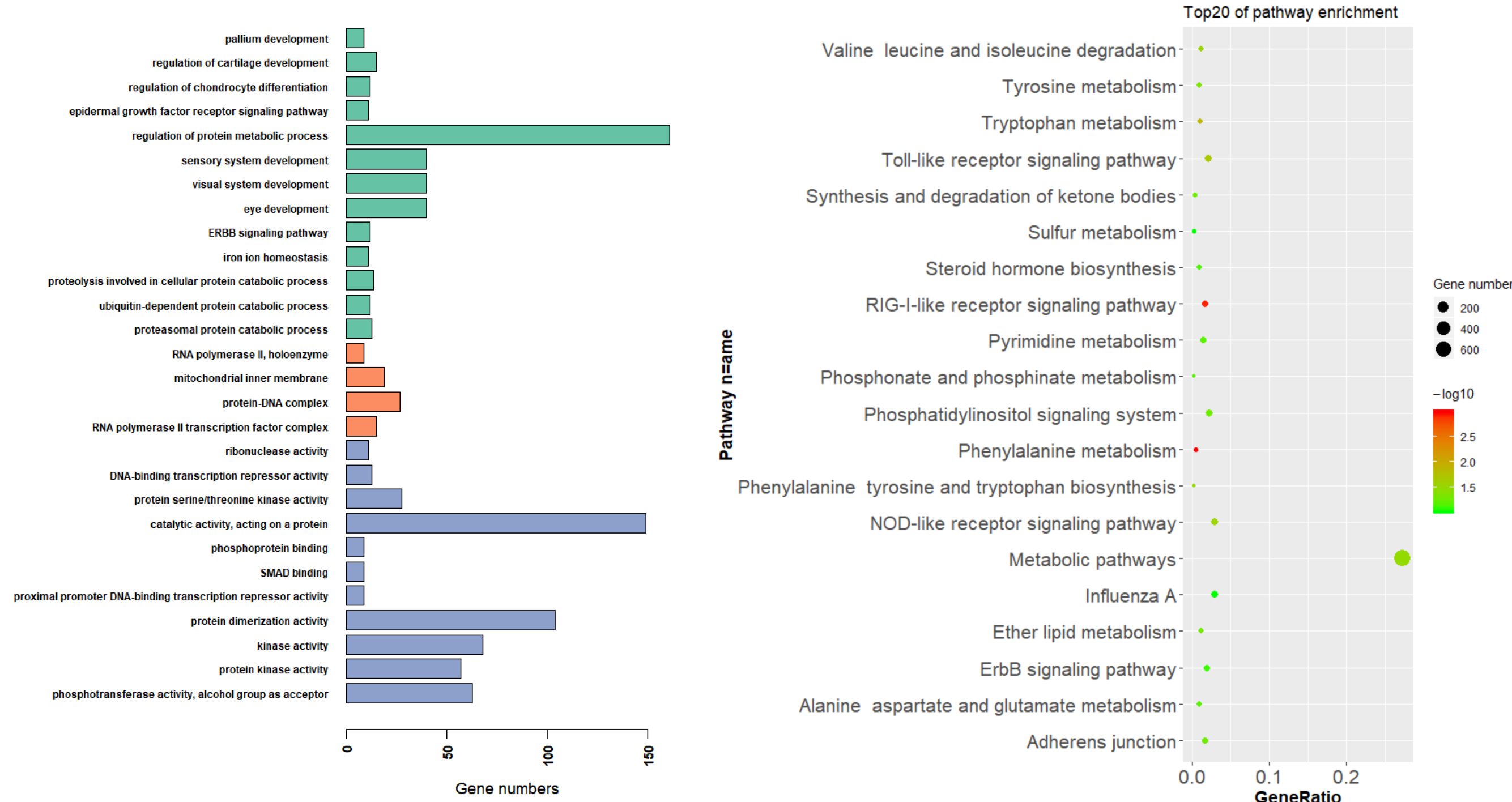

(b). Baier Yellow Chicken

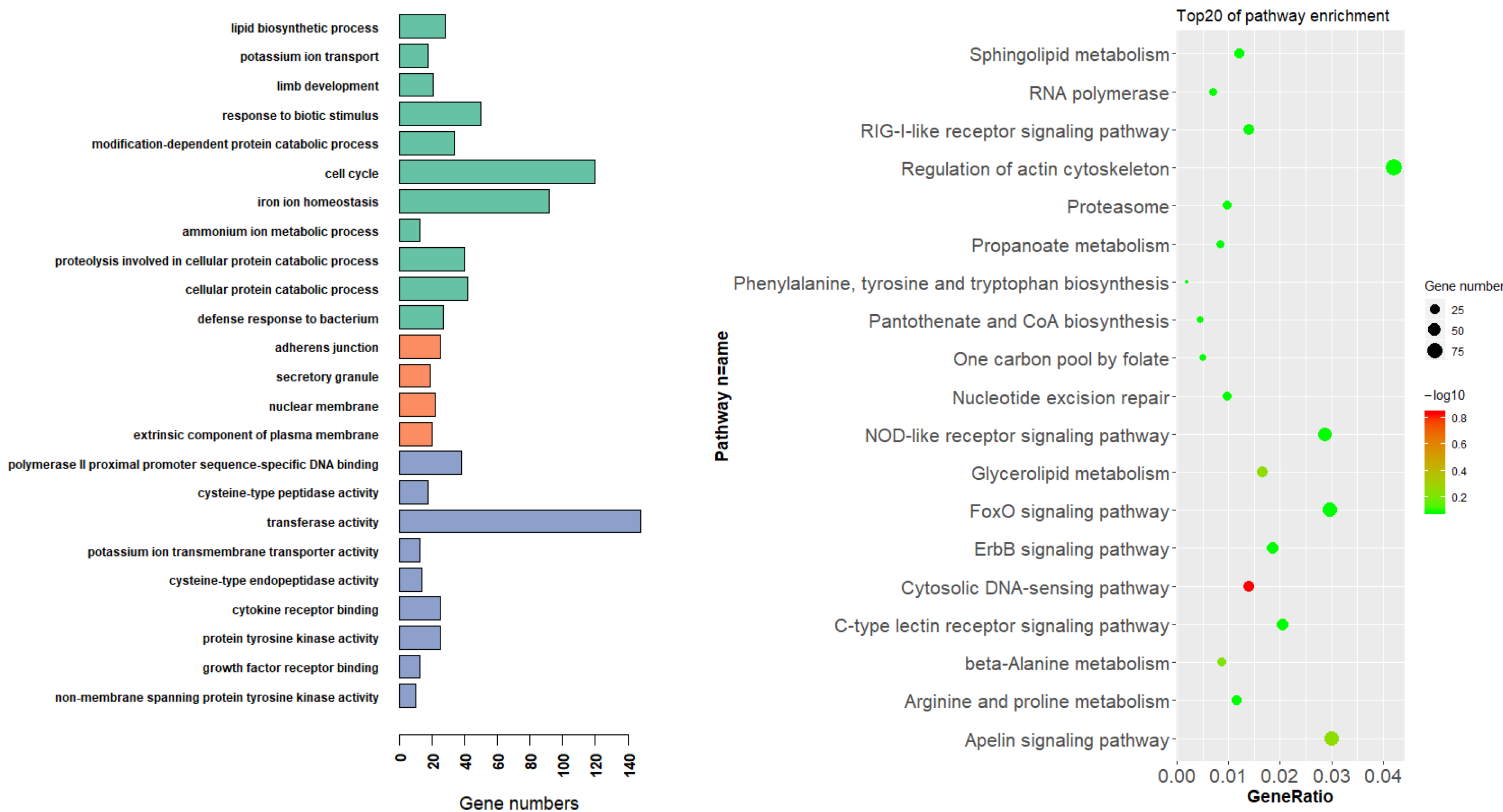

(c). Langshan Chicken

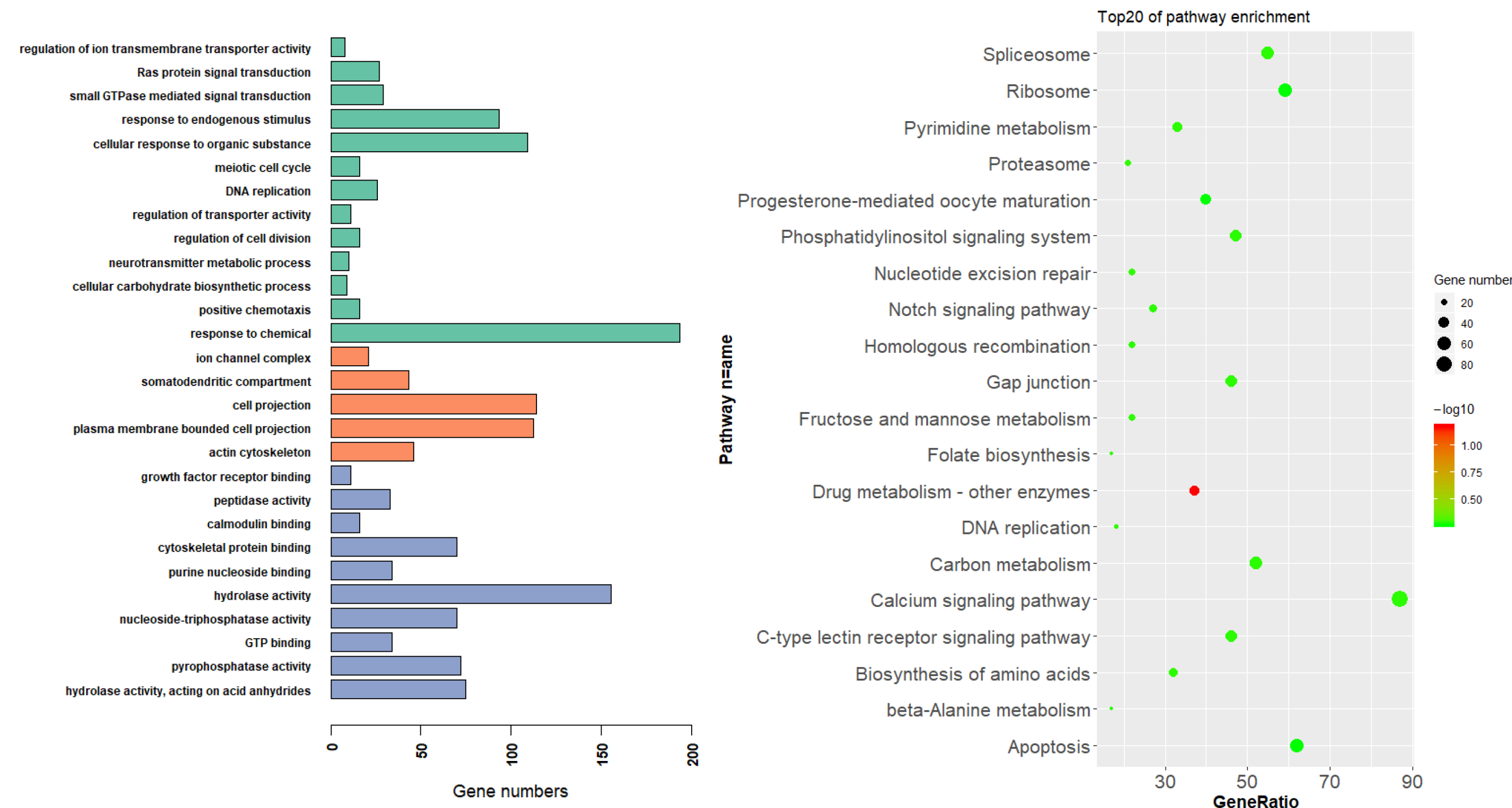

Supplement: Supplementary file 13 — Additional file 13: Figure S7. Go term and KEGG analysis for a Beijing You chicken; b Baier Yellow chicken; and c Langshan chicken. [file 12711_2023_866_MOESM13_ESM.pdf]
