# Supplementary material for: Managing genomic diversity in conservation programs of Chinese domestic chickens
Source: Genet Sel Evol. 2023 Dec 14;55:92. doi: 10.1186/s12711-023-00866-3 (PMC10722821; doi:10.1186/s12711-023-00866-3)
Supplement: Supplementary file 4 — Additional file 4: Table S2. Summary of genome sequencing and annotation of variants for the three Chinese domestic chicken breeds. [file 12711_2023_866_MOESM4_ESM.doc]

Additional file 4: Table S2. Summary of genome sequencing and annotation of variants in three Chinese domestic chicken breeds

|  | | **Number of Variants** |
| --- | --- | --- |
| **SNPs** | |  |
| 3’UTR | | 17524 |
| Intergenic | | 1071932 |
| Splicing | | 504 |
| Upstream | | 22065 |
| Exonic | Nonsynonymous | 15061 |
|  | Synonymous | 33184 |
|  | stop gain | 843 |
|  | stop loss | 37 |
| Downstream | | 32245 |
| 5’UTR | | 1904 |
| Intronic | | 1006417 |
| ncRNA | | 56914 |
| Total | | 4709111 |
| *Known SNPs* | | 3221593 |
| *Novel SNPs* | | 1487518 |
| *Novel ratio (%)* | | 31.59 |
|  | |  |
| **INDELs** | |  |
| 3’UTR | | 367 |
| Intergenic | | 185984 |
| Splicing | | 35 |
| Upstream | | 2965 |
| Exonic | stop gain | 10 |
|  | stop loss | 1 |
|  | Frameshift deletion | 367 |
|  | Frameshift insertion | 150 |
|  | Non-frameshift deletion | 125 |
|  | Non-frameshift insertion | 52 |
| Downstream | | 5223 |
| 5’UTR | | 198 |
| Intronic | | 163302 |
| Insertion | | 150 |
| Deletion | | 367 |
| Total | | 361301 |
