# Supplementary material for: Managing genomic diversity in conservation programs of Chinese domestic chickens
Source: Genet Sel Evol. 2023 Dec 14;55:92. doi: 10.1186/s12711-023-00866-3 (PMC10722821; doi:10.1186/s12711-023-00866-3)
Supplement: Supplementary file 7 — Additional file 7: Table S3. Estimation of the pairwise genetic differentiation statistic among breeds (FST). [file 12711_2023_866_MOESM7_ESM.doc]

**Additional file 7: Table S3 Estimation of the pairwise genetic differentiation statistic among breeds (Fst statistics).**

|  | BYC07 | BYC10 | BYC15 | YBYC | BEC07 | BEC10 | BEC15 | YBEC | LSC10 | LSC12 | LSC15 | YLSC |
| --- | --- | --- | --- | --- | --- | --- | --- | --- | --- | --- | --- | --- |
| BYC07 | 0 |  |  |  |  |  |  |  |  |  |  |  |
| BYC10 | 0.006958 | 0 |  |  |  |  |  |  |  |  |  |  |
| BYC15 | 0.01644 | 0.01812 | 0 |  |  |  |  |  |  |  |  |  |
| YBYC | 0.1252 | 0.1264 | 0.1379 | 0 |  |  |  |  |  |  |  |  |
| BEC07 | 0.1193 | 0.1152 | 0.1300 | 0.1271 | 0 |  |  |  |  |  |  |  |
| BEC10 | 0.1241 | 0.1197 | 0.1349 | 0.1312 | 0.004826 | 0 |  |  |  |  |  |  |
| BEC15 | 0.1370 | 0.1358 | 0.1481 | 0.1419 | 0.0334 | 0.02790 | 0 |  |  |  |  |  |
| YBEC | 0.1302 | 0.1304 | 0.1425 | 0.1137 | 0.0796 | 0.08548 | 0.09323 | 0 |  |  |  |  |
| LSC10 | 0.1298 | 0.1257 | 0.1402 | 0.1380 | 0.1139 | 0.1178 | 0.1373 | 0.1342 | 0 |  |  |  |
| LSC12 | 0.1290 | 0.1266 | 0.1406 | 0.1286 | 0.1178 | 0.1217 | 0.1357 | 0.1243 | 0.02345 | 0 |  |  |
| LSC15 | 0.1390 | 0.1373 | 0.1508 | 0.1352 | 0.1280 | 0.1319 | 0.1454 | 0.1308 | 0.04139 | 0.01911 | 0 |  |
| YLSC | 0.1372 | 0.1374 | 0.1489 | 0.1182 | 0.1266 | 0.1308 | 0.1422 | 0.1156 | 0.1069 | 0.09674 | 0.1020 | 0 |
