# Supplementary material for: Managing genomic diversity in conservation programs of Chinese domestic chickens
Source: Genet Sel Evol. 2023 Dec 14;55:92. doi: 10.1186/s12711-023-00866-3 (PMC10722821; doi:10.1186/s12711-023-00866-3)
Supplement: Supplementary file 8 — Additional file 8: Table S4. Effective population size (Ne) estimated for the three breeds in in situ and ex situ conservation programs. [file 12711_2023_866_MOESM8_ESM.doc]

Additional file 8: Table S4. Effective population sizes (Ne) for three Chinese domestic chicken breeds in in situ and ex situ conserved populations.

| Chromosome number | BEC | | | | BYC | | | | LSC | | | |
| --- | --- | --- | --- | --- | --- | --- | --- | --- | --- | --- | --- | --- |
|  | Ex situ | | | In situ | Ex situ | | | In situ | Ex situ | | | In situ |
|  | 2007 | 2010 | 2015 | 2018 | 2007 | 2010 | 2015 | 2018 | 2010 | 2012 | 2015 | 2018 |
| Chr1 | 131.4 | 104.6 | 66.3 | 167.4 | 73.8 | 136.2 | 79.5 | 91 | 133.1 | 90.2 | 140.5 | 136.8 |
| Chr2 | 120 | 91.4 | 61.2 | 150.7 | 76.1 | 123.9 | 78 | 75.2 | 94.3 | 74.2 | 111.8 | 111 |
| Chr3 | 97.9 | 91.6 | 53.8 | 117.8 | 67.3 | 110.9 | 69 | 78.3 | 108.2 | 71.7 | 110.6 | 119.2 |
| Chr4 | 93.3 | 76.4 | 36.4 | 101 | 49.3 | 74.9 | 78.3 | 76.3 | 94.2 | 77.7 | 92.1 | 111 |
| Chr5 | 80.9 | 67.4 | 37.1 | 114.5 | 49.7 | 72.7 | 48.7 | 79.9 | 104.4 | 66.4 | 75 | 87.9 |
| Chr6 | 55.9 | 43.8 | 32.6 | 64.7 | 32 | 53.8 | 32.8 | 49.3 | 79.2 | 51.7 | 48.5 | 56.1 |
| Chr7 | 52 .8 | 46.6 | 25.7 | 67.6 | 32.8 | 59.9 | 49.5 | 50.9 | 49.7 | 43.2 | 66 | 68.2 |
| Chr8 | 48.1 | 59 | 30.9 | 61.3 | 27.3 | 50.1 | 32.4 | 47.8 | 52.8 | 51.8 | 54.6 | 53.7 |
| Chr9 | 56.7 | 44.3 | 17.6 | 52.1 | 35.9 | 44.4 | 30.1 | 40 | 44.2 | 33.1 | 47.5 | 53.8 |
| Chr10 | 33.4 | 37 | 12.4 | 45.6 | 24.4 | 32.2 | 23.6 | 32.2 | 38.8 | 38.6 | 32.1 | 41 |
| Chr11 | 40.3 | 33.7 | 21.8 | 47.8 | 24.3 | 42 | 24.6 | 29.7 | 33.8 | 37.3 | 37.4 | 31.3 |
| Chr12 | 58.7 | 43 | 30.7 | 58.3 | 30.5 | 49.2 | 35.2 | 64.7 | 47.8 | 44.1 | 41.2 | 45.3 |
| Chr13 | 29 | 34.1 | 23.5 | 39.8 | 25.6 | 41.3 | 19.8 | 33.4 | 35 | 35.7 | 35.6 | 40.6 |
| Chr14 | 39.7 | 34.8 | 21 | 40.1 | 22.9 | 36.5 | 30.4 | 46.1 | 40.6 | 40.1 | 32.4 | 33.6 |
| Chr15 | 37.7 | 37.1 | 20.9 | 49.9 | 20.5 | 24 | 15.3 | 35.4 | 37.1 | 47.5 | 39.7 | 32.8 |
| Chr16 | 5.7 | 8.8 | 7.2 | 8 | 4.1 | 11.2 | 5.9 | 7.8 | 6.4 | 7.9 | 3.6 | 2.7 |
| Chr17 | 46.5 | 32.2 | 17 | 51.1 | 22.5 | 34.8 | 20.6 | 35.3 | 35.7 | 35.1 | 38.4 | 36.2 |
| Chr18 | 31.8 | 35.2 | 17.7 | 31.9 | 24.7 | 27.3 | 24.6 | 34 | 33 | 26.8 | 26.5 | 38.2 |
| Chr19 | 50.2 | 43 | 19.2 | 51.1 | 24.6 | 31.3 | 26.5 | 48.2 | 42.8 | 31.8 | 31.4 | 31.5 |
| Chr20 | 41.5 | 39.5 | 21.9 | 57.7 | 16.4 | 27.8 | 19.9 | 47.2 | 41.6 | 35.4 | 32.4 | 31.3 |
| Chr21 | 42 | 38.5 | 16.8 | 38 | 25 | 26.2 | 18.6 | 30.9 | 31 | 29.5 | 41 | 36.5 |
| Chr22 | 20.4 | 18.7 | 13 | 23.7 | 12.9 | 22.6 | 12.8 | 17.7 | 29 | 30.8 | 26.9 | 13.9 |
| Chr23 | 46.3 | 32.4 | 21.2 | 38.7 | 26.8 | 38.3 | 27.3 | 31.9 | 38.8 | 34.2 | 35.8 | 36.6 |
| Chr24 | 36.3 | 28.7 | 16.7 | 44.5 | 23.1 | 32.9 | 23.6 | 35.6 | 36.9 | 40.9 | 30.7 | 38.5 |
| Chr25 | 23.7 | 19.7 | 14.6 | 32.7 | 10.7 | 15.9 | 13.7 | 14.2 | 30.7 | 25.5 | 27.6 | 21.4 |
| Chr26 | 30 | 28.6 | 22.9 | 42.9 | 17.1 | 32.5 | 22.1 | 28 | 43.4 | 28.2 | 28.5 | 34 |
| Chr27 | 31.5 | 36.3 | 25.2 | 33.5 | 25.6 | 35.9 | 18.6 | 31.1 | 34.6 | 35.3 | 37.1 | 26.7 |
| Chr28 | 35.5 | 27.9 | 21.7 | 44.1 | 19.7 | 28.1 | 20.6 | 28.9 | 35.1 | 29.2 | 26.2 | 36.7 |
| Average (Chr 1-5) | 104.70 | 86.28 | 50.96 | 130.28 | 63.24 | 103.72 | 70.7 | 80.14 | 106.84 | 76.04 | 106 | 113.18 |
| Average (Chr 6-10) | 48.53 | 46.14 | 23.84 | 58.26 | 30.48 | 48.08 | 33.68 | 44.04 | 52.94 | 43.68 | 49.74 | 54.56 |
| Average (Chr 11-28) | 35.93 | 31.79 | 19.61 | 40.77 | 20.94 | 30.99 | 21.12 | 33.34 | 35.18 | 33.07 | 31.80 | 31.54 |
| Average (all) | 50.53 | 44.08 | 25.96 | 59.86 | 30.20 | 47.03 | 32.21 | 43.61 | 51.15 | 42.64 | 48.25 | 50.23 |
