# Supplementary material for: Managing genomic diversity in conservation programs of Chinese domestic chickens
Source: Genet Sel Evol. 2023 Dec 14;55:92. doi: 10.1186/s12711-023-00866-3 (PMC10722821; doi:10.1186/s12711-023-00866-3)
Supplement: Supplementary file 9 — Additional file 9: Table S5. Statistical summary of analysis for runs of homozygosity in in situ and ex situ conserved chicken populations. [file 12711_2023_866_MOESM9_ESM.doc]

Additional file 9: Table S5. Statistical summary for runs of homozygosity in in situ and ex situ conserved chicken populations.

|  |  | BEC | | | | BYC | | | | LSC | | | |
| --- | --- | --- | --- | --- | --- | --- | --- | --- | --- | --- | --- | --- | --- |
|  |  | Ex situ | | | In situ | Ex situ | | | In situ | Ex situ | | | In situ |
|  |  | BEC07 | BEC10 | BEC15 | YBEC | BYC07 | BYC10 | BYC15 | YBYC | LSC10 | LSC12 | LSC15 | YLSC |
| NSEG | Mean | 277 | 275.0667 | 385.9 | 240.3667 | 435.5333 | 373.3667 | 543.2333 | 401.0345 | 271.6667 | 273.6333 | 335.9667 | 372.9355 |
|  | SD | 34.43234 | 42.91044 | 92.87379 | 54.99685 | 71.34991 | 47.42797 | 76.14219 | 125.063 | 46.79842 | 39.36696 | 61.26172 | 85.29124 |
|  | Min | 197 | 212 | 203 | 143 | 246 | 296 | 370 | 251 | 215 | 200 | 209 | 181 |
|  | Max | 337 | 385 | 603 | 356 | 558 | 471 | 742 | 887 | 417 | 367 | 427 | 515 |
| KB | Mean | 52090.83 | 52664.74 | 75768.76 | 48825.05 | 86243.68 | 71543.44 | 107340.9 | 81883.66 | 50738.73 | 52909.15 | 63632.91 | 78521.53 |
|  | SD | 6510.731 | 7950.433 | 18601.42 | 11872.16 | 14934.95 | 8463.309 | 16400.86 | 28167.17 | 9911.346 | 7690.579 | 12143.71 | 19494.35 |
|  | Min | 35435 | 39065.7 | 38839.5 | 30609.4 | 53783.6 | 56933.8 | 71806.9 | 50508.9 | 39702.1 | 38422.5 | 40757.2 | 38491.5 |
|  | Max | 61448.3 | 70823.8 | 117078 | 82211.5 | 113248 | 88561.7 | 148309 | 184356 | 83345.2 | 70616.4 | 84639.5 | 111444 |
| KBAVER | Mean | 188.1392 | 191.7414 | 196.0425 | 203.0306 | 198.004 | 191.9233 | 197.3094 | 202.6213 | 186.31 | 193.4726 | 189.309 | 209.6965 |
|  | SD | 7.242002 | 8.856859 | 7.760375 | 11.11466 | 8.162784 | 5.9772 | 6.15242 | 10.89527 | 7.304308 | 7.377346 | 6.98059 | 11.613 |
|  | Min | 172.262 | 174.79 | 177.972 | 180.927 | 181.018 | 181.178 | 177.863 | 180.825 | 175.893 | 178.631 | 178.388 | 189.35 |
|  | Max | 203.169 | 208.118 | 215.295 | 230.931 | 218.633 | 203.426 | 211.074 | 226.847 | 200.155 | 206.857 | 205.798 | 233.681 |
| NSNP | Mean | 81.6781 | 82.39433 | 87.70588 | 82.72154 | 87.8829 | 83.68146 | 89.87311 | 88.05821 | 80.25472 | 81.11999 | 83.13712 | 91.07032 |
|  | SD | 30.3393 | 29.58904 | 35.90122 | 30.76624 | 36.40695 | 31.59871 | 37.86531 | 36.63116 | 29.44159 | 30.21881 | 31.47814 | 40.37923 |
|  | Min | 50 | 50 | 50 | 50 | 50 | 50 | 50 | 50 | 50 | 50 | 50 | 50 |
|  | Max | 344 | 385 | 386 | 354 | 448 | 381 | 539 | 478 | 355 | 350 | 465 | 534 |
| Density | Mean | 2.386938 | 2.407329 | 2.340044 | 2.563433 | 2.344431 | 2.375111 | 2.289537 | 2.426634 | 2.407817 | 2.467005 | 2.36736 | 2.432933 |
|  | SD | 1.643706 | 1.716774 | 1.660818 | 2.196482 | 1.559804 | 1.572183 | 1.374075 | 2.129117 | 1.485549 | 1.864891 | 1.500665 | 2.022848 |
|  | Min | 0.786 | 0.764 | 0.804 | 0.742 | 0.672 | 0.817 | 0.764 | 0.707 | 0.823 | 0.838 | 0.737 | 0.762 |
|  | Max | 34.363 | 32.869 | 36.889 | 44.888 | 39.515 | 38.895 | 27.386 | 45.457 | 33.275 | 43.695 | 43.695 | 31.896 |
| PHOM | Mean | 0.982111 | 0.982054 | 0.982564 | 0.98167 | 0.982673 | 0.982036 | 0.982801 | 0.982226 | 0.982084 | 0.981943 | 0.982163 | 0.982478 |
|  | SD | 0.007258 | 0.007239 | 0.007331 | 0.017691 | 0.007349 | 0.007326 | 0.007272 | 0.00721 | 0.007312 | 0.007394 | 0.007513 | 0.019451 |
