# Supplementary material for: Managing genomic diversity in conservation programs of Chinese domestic chickens
Source: Genet Sel Evol. 2023 Dec 14;55:92. doi: 10.1186/s12711-023-00866-3 (PMC10722821; doi:10.1186/s12711-023-00866-3)
Supplement: Supplementary file 11 — Additional file 11: Table S6. Candidate regions in a Baier Yellow chicken; b Beijing You chicken; and c Langshan chicken. [file 12711_2023_866_MOESM11_ESM.doc]

Additional file 11: Table S6. Candidate overlapped regions selected by three methods

Beijing You Chicken

| Chr | Region | Log2Pi | F_ST_ | XP-EHH |
| --- | --- | --- | --- | --- |
| 1 | 50020001-50260000 | 1.38451612 | 0.110285286 | 2.024214286 |
| 1 | 63890001-64070000 | 1.826697021 | 0.089294243 | 2.43347 |
| 1 | 104350001-104490000 | 1.391238616 | 0.1246976 | 2.18846 |
| 1 | 125690001-125810000 | 1.579991064 | 0.117003333 | 2.58553 |
| 1 | 141940001-142220000 | 2.16023522 | 0.106184042 | 2.18688 |
| 1 | 148400001-148550000 | 2.538510335 | 0.119847167 | 2.4446 |
| 1 | 148610001-149020000 | 1.905944351 | 0.134416893 | 2.46476 |
| 1 | 150880001-151120000 | 1.934209732 | 0.141194857 | 2.01074 |
| 1 | 160600001-160720000 | 1.741760955 | 0.091430933 | 1.99173 |
| 1 | 164400001-164530000 | 1.46625199 | 0.16055875 | 2.03136 |
| 1 | 164860001-164960000 | 1.255227948 | 0.114957 | 2.24799 |
| 1 | 184860001-184960000 | 1.323774894 | 0.104042 | 2.16397 |
| 1 | 191560001-191900000 | 1.764293115 | 0.093957324 | 2.07593 |
| 1 | 194700001-194840000 | 1.344010475 | 0.1457218 | 2.24991 |
| 2 | 36580001-36690000 | 1.386052735 | 0.1812615 | 2.17577 |
| 2 | 36960001-37150000 | 1.636103585 | 0.1671813 | 2.042232 |
| 2 | 76450001-76580000 | 1.631830768 | 0.11373175 | 2.09256 |
| 2 | 78010001-78170000 | 1.636224323 | 0.131983429 | 2.2142 |
| 2 | 88220001-88340000 | 1.455167706 | 0.110123667 | 2.11674 |
| 2 | 88440001-88590000 | 1.606579652 | 0.093241733 | 2.409141667 |
| 2 | 91890001-92030000 | 1.867194809 | 0.104023 | 2.05055 |
| 2 | 92780001-92930000 | 1.638316565 | 0.161216333 | 2.25499 |
| 2 | 93840001-94020000 | 1.803591262 | 0.099016311 | 2.0453 |
| 2 | 95860001-96070000 | 2.084336029 | 0.137042333 | 2.44398 |
| 2 | 101730001-101890000 | 1.781747798 | 0.165224429 | 2.09091 |
| 2 | 102260001-102430000 | 2.205457302 | 0.184260625 | 1.9786075 |
| 2 | 104970001-105070000 | 2.645867901 | 0.126129 | 2.23033 |
| 2 | 105780001-106070000 | 1.681801271 | 0.139566718 | 2.35372 |
| 2 | 107000001-107110000 | 1.254704209 | 0.1178795 | 2.12268 |
| 2 | 107180001-107430000 | 1.47214896 | 0.086846327 | 2.157167333 |
| 2 | 117780001-117880000 | 1.567763276 | 0.135221 | 2.34469 |
| 2 | 119830001-119930000 | 1.311742701 | 0.142257 | 2.27472 |
| 2 | 126710001-126810000 | 1.29298255 | 0.130526 | 2.33507 |
| 2 | 127030001-127160000 | 1.533515783 | 0.135294 | 2.29226 |
| 2 | 134440001-134610000 | 1.587676294 | 0.0575431 | 2.28001 |
| 2 | 143120001-143290000 | 2.37316692 | 0.130219875 | 2.1996 |
| 2 | 144580001-144680000 | 1.473858323 | 0.124221186 | 1.991561429 |
| 3 | 20090001- 20240000 | 1.509308509 | 0.1283665 | 1.96777 |
| 3 | 21640001-21930000 | 1.481420881 | 0.114736283 | 2.020636667 |
| 3 | 22090001-22250000 | 1.275882983 | 0.122986667 | 2.261356667 |
| 3 | 22270001-22430000 | 1.761306527 | 0.127601143 | 2.33087 |
| 3 | 26280001-26420000 | 1.50476777 | 0.1187026 | 2.22261 |
| 3 | 30330001-30480000 | 1.922004205 | 0.093644117 | 1.9707 |
| 3 | 46720001-46830000 | 1.50352656 | 0.10011325 | 2.24535 |
| 3 | 47430001-47670000 | 1.346968788 | 0.104329367 | 2.083075 |
| 3 | 49190001-49320000 | 1.666895806 | 0.1970335 | 2.08951 |
| 3 | 50210001-50360000 | 1.365041927 | 0.115998833 | 2.25795 |
| 3 | 56040001-56250000 | 2.482268744 | 0.133593667 | 1.97299 |
| 3 | 57790001-57910000 | 1.482143555 | 0.094374133 | 2.02726 |
| 3 | 62190001-62290000 | 1.348101752 | 0.0878292 | 2.63558 |
| 3 | 62630001-62860000 | 1.616797258 | 0.1227885 | 2.10753 |
| 3 | 94120001-94270000 | 1.549178337 | 0.1361625 | 2.290115 |
| 4 | 20680001-20790000 | 1.385491477 | 0.09844285 | 2.21597 |
| 4 | 31110001-31300000 | 1.703742314 | 0.1298451 | 2.20084 |
| 4 | 33100001-33290000 | 1.655420117 | 0.1333929 | 2.34897 |
| 4 | 34390001-34500000 | 1.325257297 | 0.1269605 | 2.14201 |
| 4 | 38080001-38270000 | 1.561641877 | 0.121767275 | 2.344285 |
| 4 | 39170001-39270000 | 1.28360078 | 0.114717 | 2.23731 |
| 4 | 39320001-39530000 | 1.806657501 | 0.124636417 | 2.653515 |
| 4 | 39630001-39800000 | 1.778221986 | 0.13467975 | 2.3322425 |
| 4 | 39890001-40060000 | 1.558307551 | 0.105153371 | 2.26426 |
| 4 | 42070001-42180000 | 1.565928093 | 0.106804 | 2.58874 |
| 4 | 43510001-43820000 | 1.866000299 | 0.103650925 | 2.547898125 |
| 4 | 43970001-44700000 | 1.657591127 | 0.134387481 | 2.311729038 |
| 4 | 44850001-45320000 | 1.609782778 | 0.119649185 | 2.014139412 |
| 4 | 45370001-45470000 | 1.472240155 | 0.1316 | 2.92543 |
| 4 | 45480001-45660000 | 1.52336391 | 0.1381504 | 2.1983 |
| 4 | 45830001-45940000 | 1.288738105 | 0.1080115 | 2.1413 |
| 4 | 47630001-47760000 | 1.773855027 | 0.120724 | 1.99196 |
| 4 | 48500001-48610000 | 1.424766373 | 0.141747 | 2.21974 |
| 4 | 50580001-50680000 | 1.315530044 | 0.104343 | 2.24931 |
| 4 | 57490001-57730000 | 1.863363907 | 0.102524747 | 2.182180667 |
| 4 | 63400001-63530000 | 1.433067715 | 0.133162333 | 2.0907 |
| 4 | 70400001-70500000 | 1.26052297 | 0.0897509 | 2.72241 |
| 4 | 73850001-73990000 | 1.298890355 | 0.115707333 | 2.07748 |
| 5 | 640001-800000 | 1.410094218 | 0.144383286 | 2.44068 |
| 5 | 5440001-5560000 | 1.3274181 | 0.129488333 | 1.96637 |
| 5 | 5670001-5770000 | 1.259876203 | 0.161007 | 1.96637 |
| 5 | 21690001-21860000 | 1.542673893 | 0.12834025 | 2.09107 |
| 5 | 23480001-23690000 | 1.538697854 | 0.089559627 | 2.10522 |
| 5 | 24160001-24290000 | 1.380472546 | 0.131333 | 2.03062 |
| 5 | 25620001-25880000 | 2.000503212 | 0.140752471 | 2.062703529 |
| 5 | 26280001-26460000 | 1.417843802 | 0.103141833 | 2.31892 |
| 5 | 29850001-30030000 | 1.616548098 | 0.124110222 | 2.00497 |
| 5 | 30820001-31310000 | 3.587607701 | 0.1966227 | 2.25077275 |
| 5 | 31800001-31940000 | 1.823044466 | 0.1184414 | 2.285 |
| 5 | 33680001-33780000 | 1.433020198 | 0.153856 | 2.00847 |
| 5 | 46840001-47070000 | 1.670204103 | 0.127290214 | 2.029834286 |
| 5 | 49960001-50110000 | 1.484026199 | 0.125669167 | 2.06544 |
| 5 | 54720001-54860000 | 1.41739969 | 0.12410525 | 2.07687 |
| 5 | 54890001-55070000 | 1.687685687 | 0.185928 | 2.031736667 |
| 5 | 55720001-55910000 | 1.351931313 | 0.155847111 | 2.203386667 |
| 5 | 58160001-58360000 | 1.813869488 | 0.140968364 | 2.111745455 |
| 5 | 58440001-58680000 | 1.987531639 | 0.099602492 | 2.201135385 |
| 6 | 18520001-18700000 | 2.263401044 | 0.143885333 | 2.48894 |
| 6 | 18970001-19090000 | 1.283883869 | 0.1151615 | 2.10884 |
| 6 | 19120001-19460000 | 1.526725668 | 0.117215513 | 2.226207333 |
| 6 | 20160001-20290000 | 1.277295007 | 0.13466175 | 2.3192 |
| 6 | 21690001-21820000 | 1.2908488 | 0.149656667 | 2.04453 |
| 7 | 2330001-2510000 | 2.200967146 | 0.167163 | 1.99959 |
| 7 | 3760001-3890000 | 1.397594061 | 0.1172035 | 1.97313 |
| 7 | 5780001-5930000 | 1.535910841 | 0.1533795 | 2.00895 |
| 7 | 6770001-7050000 | 2.210002126 | 0.171417278 | 2.26184 |
| 7 | 7070001-7280000 | 1.742500088 | 0.1949662 | 2.222348 |
| 7 | 28960001-29080000 | 1.686226392 | 0.111066 | 1.96805 |
| 7 | 29840001-30020000 | 1.512681168 | 0.105043413 | 2.1188375 |
| 7 | 35380001-35560000 | 1.721996634 | 0.113393 | 1.99858 |
| 7 | 35760001-35960000 | 2.067998296 | 0.109461727 | 2.226265455 |
| 8 | 1560001-1710000 | 1.67253341 | 0.169554833 | 2.09675 |
| 8 | 12190001-12370000 | 1.975003235 | 0.131556 | 2.06612 |
| 8 | 17900001-18050000 | 1.461129104 | 0.13184 | 2.27007 |
| 8 | 21050001-21210000 | 1.478960151 | 0.1291865 | 2.17558 |
| 8 | 23660001-23820000 | 1.938216219 | 0.107681571 | 1.9849 |
| 9 | 6350001-6500000 | 1.417285845 | 0.134945667 | 2.47925 |
| 9 | 10920001-11060000 | 1.772107806 | 0.147431 | 2.25264 |
| 9 | 23900001-24010000 | 1.32066135 | 0.129465 | 2.29476 |
| 10 | 540001-710000 | 1.54060048 | 0.118052713 | 2.07996 |
| 10 | 2690001-2810000 | 1.697612567 | 0.115321 | 2.00321 |
| 10 | 5710001-5840000 | 1.435815217 | 0.1016311 | 2.14379 |
| 10 | 15990001-16190000 | 2.286296252 | 0.166839636 | 1.9849 |
| 10 | 19600001-19790000 | 2.034295734 | 0.1911687 | 2.01033 |
| 11 | 5690001-5790000 | 1.276885708 | 0.118438 | 2.13488 |
| 11 | 9210001-9330000 | 1.32920749 | 0.11775 | 2.35083 |
| 11 | 9440001-9540000 | 1.244303508 | 0.101028 | 2.35083 |
| 11 | 12860001-12960000 | 1.270249204 | 0.117652 | 2.50924 |
| 11 | 12990001-13200000 | 1.694789823 | 0.1352275 | 2.50924 |
| 11 | 13340001-13440000 | 1.247181556 | 0.116194 | 2.93155 |
| 11 | 13450001-13570000 | 1.338924374 | 0.113491333 | 2.384063333 |
| 12 | 2520001-2630000 | 1.35940212 | 0.0994527 | 2.27561 |
| 12 | 2770001-2970000 | 1.577024786 | 0.1590695 | 2.028458 |
| 12 | 4950001-5980000 | 2.306942382 | 0.140569978 | 3.246457957 |
| 12 | 5990001-6350000 | 1.954371482 | 0.12103072 | 3.0333128 |
| 12 | 7330001-7430000 | 1.240115959 | 0.134129 | 2.01351 |
| 12 | 8050001-8190000 | 2.32238246 | 0.15715 | 2.053426 |
| 12 | 8320001-8510000 | 1.548446077 | 0.123405 | 2.18023 |
| 12 | 8870001-8990000 | 1.289031187 | 0.129947333 | 2.00217 |
| 12 | 11550001-11730000 | 1.593975538 | 0.124678444 | 2.20046 |
| 13 | 3800001-3950000 | 1.680646337 | 0.102976 | 1.9828 |
| 13 | 6140001-6400000 | 1.81155901 | 0.131623818 | 2.15565 |
| 13 | 13600001-13720000 | 1.326974105 | 0.107226 | 1.98715 |
| 14 | 4110001-4230000 | 1.463347244 | 0.147437 | 2.26629 |
| 14 | 12650001-12770000 | 2.059861954 | 0.153344 | 2.0454 |
| 15 | 530001-840000 | 1.455174419 | 0.127553071 | 2.37224 |
| 15 | 950001-1110000 | 1.251812768 | 0.1468005 | 2.10747 |
| 15 | 5230001-5330000 | 1.334235668 | 0.0886592 | 2.0085 |
| 15 | 9340001-9450000 | 1.371152379 | 0.118732 | 2.15699 |
| 15 | 11140001-11290000 | 1.584925269 | 0.148055 | 2.38124 |
| 15 | 11550001-11680000 | 1.243823029 | 0.0947942 | 2.30559 |
| 1 | 6450001-6550000 | 7.326222817 | 0.125254 | 1.97421 |
| 1 | 7450001-7560000 | 7.814731602 | 0.09479145 | 2.07497 |
| 17 | 3060001-3250000 | 1.840296801 | 0.1301538 | 2.28656 |
| 17 | 3430001-3540000 | 1.657812002 | 0.10299 | 2.3037 |
| 18 | 1370001-1480000 | 1.507806534 | 0.154937 | 1.9811 |
| 18 | 2080001-2190000 | 1.346453433 | 0.122876 | 1.99338 |
| 18 | 6250001-6400000 | 1.67495633 | 0.130593833 | 2.121215 |
| 20 | 6410001-6520000 | 1.311678392 | 0.1430005 | 2.15744 |
| 20 | 12730001-12860000 | 1.475021236 | 0.14028575 | 2.25085 |
| 21 | 440001-610000 | 1.320871799 | 0.126178857 | 2.23969 |
| 21 | 770001-990000 | 1.865104664 | 0.161903385 | 2.07527 |
| 21 | 1410001-1590000 | 1.737326663 | 0.107085556 | 2.039891111 |
| 21 | 4660001-4780000 | 1.340988234 | 0.105605 | 1.98655 |
| 23 | 1860001-2000000 | 1.458326697 | 0.1040684 | 2.16045 |
| 24 | 1680001-1820000 | 1.402992281 | 0.1108058 | 1.99063 |
| 25 | 2430001-2680000 | 1.995060875 | 0.0994148 | 1.9959275 |
| 26 | 620001-740000 | 1.383479883 | 0.100727 | 2.03101 |
| 26 | 970001-1150000 | 1.621988797 | 0.100338 | 2.090091111 |
| 26 | 1240001-1370000 | 3.770048843 | 0.0968204 | 2.8408425 |
| 27 | 4590001-4790000 | 1.929221464 | 0.135070091 | 1.96853 |
| 28 | 90001-470000 | 1.532332643 | 0.132419 | 2.54213 |
| 28 | 740001-840000 | 1.392398233 | 0.102227 | 2.13335 |
| 28 | 3510001-3620000 | 1.290008331 | 0.1132855 | 2.50195 |
| 28 | 3790001-4030000 | 1.404085665 | 0.131147375 | 2.27405125 |
| 28 | 4340001-4530000 | 1.546608763 | 0.1414598 | 2.202625 |
| Z | 1420001-1590000 | 1.602792494 | 0.1003866 | 2.41805 |
| Z | 1930001-2080000 | 2.551991442 | 0.143781 | 2.25 |
| Z | 3010001-3460000 | 1.782479317 | 0.135198063 | 2.08547375 |
| Z | 3470001-3600000 | 3.259703 | 0.0990991 | 2.9079525 |
| Z | 9610001-9800000 | 2.688701284 | 0.1305622 | 2.09782 |
| Z | 11490001-11730000 | 1.99564774 | 0.166948 | 2.86522 |
| Z | 34360001-34610000 | 1.576299534 | 0.084173269 | 1.97343 |
| Z | 48410001-48510000 | 1.387032395 | 0.0919015 | 2.23981 |
| Z | 54190001-54410000 | 1.758485434 | 0.168336615 | 1.99056 |
| Z | 54510001-54640000 | 3.026012343 | 0.106421 | 2.33378 |
| Z | 77550001-77720000 | 2.406739713 | 0.15489 | 2.21569 |
| Z | 79480001-79750000 | 1.805027135 | 0.118175571 | 1.98713 |
| Z | 81330001-81430000 | 1.805027135 | 0.118175571 | 1.98713 |

Langshan chicken

| Chr | Region | | Log2Pi | F_ST_ | XP-EHH |
| --- | --- | --- | --- | --- | --- |
| 1 | 330001 | 450000 | 1.644344241 | 0.125722 | 2.01953 |
| 1 | 10020001 | 10120000 | 0.837893 | 0.103995 | 2.23006 |
| 1 | 61410001 | 61510000 | 0.894638 | 0.084548 | 2.29537 |
| 1 | 61540001 | 61670000 | 1.001814 | 0.100984 | 2.19449 |
| 1 | 61910001 | 62350000 | 1.027612 | 0.086826 | 2.277577 |
| 1 | 63720001 | 63950000 | 1.326283 | 0.085441 | 2.429369 |
| 1 | 103160001 | 103460000 | 1.376608 | 0.089691 | 2.13669 |
| 1 | 104300001 | 104400000 | 0.85772 | 0.116011 | 2.01848 |
| 1 | 106860001 | 107040000 | 0.946226 | 0.084969 | 2.278647 |
| 1 | 109510001 | 109670000 | 0.97113 | 0.111472 | 2.133083 |
| 1 | 129230001 | 129610000 | 1.171449 | 0.12888 | 2.199442 |
| 1 | 129630001 | 129730000 | 1.154865 | 0.080379 | 1.97181 |
| 1 | 130430001 | 130550000 | 0.911048 | 0.061453 | 2.17942 |
| 1 | 130580001 | 130710000 | 1.084903 | 0.073169 | 2.854363 |
| 1 | 131770001 | 131900000 | 0.925773 | 0.118162 | 2.32522 |
| 1 | 141980001 | 142080000 | 0.906999 | 0.112926 | 2.00275 |
| 1 | 143550001 | 143690000 | 0.926248 | 0.090658 | 2.20061 |
| 1 | 148060001 | 148220000 | 1.421594 | 0.119843 | 2.138533 |
| 1 | 148300001 | 148440000 | 1.546192 | 0.105029 | 2.234058 |
| 1 | 149190001 | 149340000 | 0.914026 | 0.08014 | 2.01313 |
| 1 | 158660001 | 158800000 | 1.090105 | 0.141455 | 2.27951 |
| 1 | 160760001 | 160940000 | 1.03986 | 0.133979 | 2.04214 |
| 1 | 161130001 | 161320000 | 1.126876 | 0.14021 | 1.99416 |
| 1 | 163430001 | 163560000 | 0.894468 | 0.185869 | 1.97628 |
| 1 | 165050001 | 165310000 | 0.95564 | 0.10647 | 2.09217 |
| 1 | 173630001 | 173840000 | 0.997961 | 0.105828 | 2.534644 |
| 1 | 193650001 | 193800000 | 1.222701 | 0.103596 | 2.13822 |
| 1 | 194190001 | 194330000 | 0.938258 | 0.127424 | 2.27443 |
| 1 | 194700001 | 194900000 | 1.238772 | 0.138057 | 2.17054 |
| 2 | 36260001 | 36480000 | 1.155467 | 0.131338 | 2.19988 |
| 2 | 36950001 | 37150000 | 1.224562 | 0.193529 | 1.97553 |
| 2 | 37360001 | 37610000 | 0.998577 | 0.122149 | 2.23733 |
| 2 | 67010001 | 67180000 | 1.022557 | 0.107927 | 2.031409 |
| 2 | 85540001 | 85710000 | 1.338623 | 0.092302 | 2.15419 |
| 2 | 90960001 | 91060000 | 1.019693 | 0.09252 | 2.06084 |
| 2 | 95920001 | 96200000 | 1.631029 | 0.086725 | 2.0877 |
| 2 | 102850001 | 102950000 | 0.836396 | 0.105978 | 2.0944 |
| 2 | 120320001 | 120730000 | 1.445959 | 0.126681 | 2.06739 |
| 2 | 120920001 | 121090000 | 1.649007 | 0.196275 | 2.007121 |
| 2 | 122920001 | 123060000 | 0.944814 | 0.120756 | 2.07216 |
| 2 | 127040001 | 127150000 | 0.868938 | 0.102017 | 2.0333 |
| 2 | 138050001 | 138150000 | 0.846505 | 0.09145 | 2.83752 |
| 2 | 138370001 | 138480000 | 0.904791 | 0.113336 | 2.07955 |
| 3 | 17800001 | 17920000 | 1.060503 | 0.105977 | 2.164 |
| 3 | 19340001 | 19440000 | 0.838995 | 0.085213 | 2.94288 |
| 3 | 22300001 | 22400000 | 0.902487 | 0.094615 | 1.96027 |
| 3 | 28320001 | 28510000 | 1.116428 | 0.116252 | 2.03589 |
| 3 | 35170001 | 35430000 | 1.604867 | 0.122266 | 2.084791 |
| 3 | 41460001 | 41640000 | 1.498724 | 0.090123 | 2.11799 |
| 3 | 43590001 | 43780000 | 1.147526 | 0.123724 | 2.17286 |
| 3 | 44280001 | 44420000 | 0.983829 | 0.124485 | 2.10783 |
| 3 | 52890001 | 53040000 | 0.891462 | 0.118047 | 1.96048 |
| 3 | 58310001 | 58500000 | 1.001814 | 0.078782 | 2.24152 |
| 3 | 58570001 | 58880000 | 1.053136 | 0.09432 | 2.482375 |
| 3 | 59790001 | 59930000 | 0.895289 | 0.103876 | 2.07527 |
| 3 | 60410001 | 60510000 | 1.810968 | 0.192204 | 2.03637 |
| 3 | 64780001 | 64990000 | 1.265009 | 0.09773 | 2.53163 |
| 3 | 67070001 | 67240000 | 1.350622 | 0.134832 | 2.09051 |
| 3 | 70260001 | 70520000 | 1.125996 | 0.097605 | 2.03376 |
| 3 | 80000001 | 80150000 | 1.388709 | 0.05524 | 2.34932 |
| 3 | 81410001 | 81550000 | 0.971165 | 0.107586 | 2.00309 |
| 3 | 83070001 | 83380000 | 1.05028 | 0.123223 | 2.124348 |
| 3 | 84020001 | 84150000 | 0.910868 | 0.071338 | 2.25412 |
| 3 | 84690001 | 84790000 | 1.128862 | 0.070753 | 3.3211 |
| 3 | 84800001 | 84990000 | 2.015908 | 0.117603 | 3.3211 |
| 3 | 85630001 | 85920000 | 1.183467 | 0.136215 | 2.00948 |
| 3 | 86670001 | 86880000 | 1.058983 | 0.100024 | 2.05922 |
| 3 | 89930001 | 90170000 | 1.06024 | 0.098102 | 2.29501 |
| 3 | 92980001 | 93140000 | 1.115579 | 0.09729 | 1.9851 |
| 3 | 93300001 | 93480000 | 1.066033 | 0.121335 | 2.47849 |
| 3 | 97510001 | 97640000 | 1.034137 | 0.118331 | 2.13605 |
| 3 | 97890001 | 98270000 | 1.590126 | 0.094655 | 2.50655 |
| 3 | 98470001 | 98600000 | 1.112655 | 0.129472 | 2.383368 |
| 3 | 100080001 | 100260000 | 1.127422 | 0.108548 | 2.04872 |
| 3 | 101260001 | 101410000 | 1.077642 | 0.13053 | 2.00715 |
| 3 | 102280001 | 102400000 | 0.90285 | 0.129411 | 2.03251 |
| 3 | 103220001 | 103440000 | 0.901365 | 0.084448 | 2.04052 |
| 3 | 109880001 | 109990000 | 0.967264 | 0.084682 | 2.42709 |
| 4 | 2680001 | 2860000 | 0.924772 | 0.089515 | 2.06588 |
| 4 | 4270001 | 4450000 | 0.886681 | 0.096875 | 2.02323 |
| 4 | 14460001 | 14620000 | 0.906127 | 0.091496 | 2.11968 |
| 4 | 14930001 | 15050000 | 1.083512 | 0.059638 | 2.52591 |
| 4 | 17940001 | 18100000 | 1.015565 | 0.154884 | 1.98911 |
| 4 | 20440001 | 20670000 | 1.100491 | 0.114356 | 2.18383 |
| 4 | 20740001 | 21020000 | 1.267885 | 0.147822 | 2.18043 |
| 4 | 28770001 | 28880000 | 0.873615 | 0.144911 | 2.28814 |
| 4 | 63700001 | 63890000 | 1.130126 | 0.077515 | 2.22904 |
| 4 | 64320001 | 64430000 | 1.256475 | 0.102901 | 2.14897 |
| 4 | 68480001 | 68680000 | 1.201371 | 0.090047 | 1.97145 |
| 4 | 70150001 | 70260000 | 0.934934 | 0.080292 | 2.07556 |
| 4 | 70320001 | 70440000 | 1.737266 | 0.06935 | 2.07556 |
| 4 | 72290001 | 72440000 | 1.159415 | 0.145637 | 2.03698 |
| 4 | 73190001 | 73440000 | 1.386664 | 0.149132 | 2.19083 |
| 4 | 81790001 | 81940000 | 0.96316 | 0.098956 | 2.01708 |
| 4 | 88810001 | 88920000 | 1.000646 | 0.090944 | 2.09269 |
| 5 | 400001 | 540000 | 0.934957 | 0.099736 | 2.20909 |
| 5 | 780001 | 1050000 | 0.926125 | 0.080419 | 2.423555 |
| 5 | 10590001 | 10710000 | 1.000517 | 0.088475 | 2.62914 |
| 5 | 27270001 | 27380000 | 1.039702 | 0.084735 | 2.1246 |
| 5 | 28810001 | 29090000 | 1.218754 | 0.097373 | 2.00535 |
| 5 | 30770001 | 30960000 | 1.324565 | 0.091846 | 2.4651 |
| 5 | 32690001 | 32820000 | 0.90383 | 0.096537 | 2.04389 |
| 5 | 33010001 | 33160000 | 1.106661 | 0.101118 | 2.357398 |
| 5 | 34260001 | 34370000 | 0.857697 | 0.093552 | 2.025355 |
| 5 | 34770001 | 35060000 | 1.160975 | 0.093154 | 2.573741 |
| 5 | 35780001 | 35880000 | 0.838426 | 0.089386 | 1.99382 |
| 5 | 37270001 | 37440000 | 1.034995 | 0.082446 | 2.32251 |
| 5 | 38260001 | 38360000 | 0.986735 | 0.108885 | 2.32496 |
| 5 | 38380001 | 38560000 | 1.107292 | 0.099605 | 2.318481 |
| 5 | 38770001 | 38990000 | 1.07319 | 0.128246 | 2.19676 |
| 5 | 39000001 | 39250000 | 1.821325 | 0.100265 | 2.669685 |
| 5 | 43190001 | 43300000 | 0.979697 | 0.109387 | 1.97281 |
| 5 | 45590001 | 45750000 | 0.970692 | 0.119323 | 2.65791 |
| 5 | 48130001 | 48230000 | 0.968337 | 0.103751 | 2.00721 |
| 5 | 51670001 | 52000000 | 1.012581 | 0.118122 | 2.251977 |
| 6 | 15540001 | 15710000 | 1.215377 | 0.094337 | 2.02044 |
| 6 | 16540001 | 16700000 | 1.463965 | 0.110131 | 1.99143 |
| 6 | 22690001 | 22980000 | 1.135878 | 0.113995 | 2.39511 |
| 6 | 23040001 | 23430000 | 1.521129 | 0.142979 | 2.448648 |
| 6 | 26240001 | 26380000 | 1.463654 | 0.123816 | 2.38086 |
| 6 | 26490001 | 26680000 | 1.615337 | 0.109736 | 2.174963 |
| 6 | 28540001 | 1130000 | 0.899214 | 0.094284 | 2.497353 |
| 7 | 8920001 | 9200000 | 1.4246 | 0.116363 | 2.24865 |
| 7 | 11540001 | 11680000 | 1.096177 | 0.112518 | 2.26305 |
| 7 | 11860001 | 12020000 | 1.0005 | 0.130959 | 2.51042 |
| 7 | 28040001 | 28150000 | 0.958584 | 0.106466 | 2.08633 |
| 7 | 29020001 | 29140000 | 0.896645 | 0.084221 | 2.02349 |
| 7 | 29410001 | 29550000 | 0.941546 | 0.102511 | 2.2972 |
| 8 | 1380001 | 1570000 | 1.046844 | 0.09241 | 2.25691 |
| 8 | 20400001 | 1410000 | 0.862263 | 0.103494 | 2.035148 |
| 9 | 6510001 | 6770000 | 1.862833 | 0.112211 | 2.108226 |
| 9 | 22060001 | 22220000 | 0.917618 | 0.093835 | 2.076988 |
| 10 | 670001 | 820000 | 0.945818 | 0.130386 | 1.98721 |
| 10 | 2630001 | 2760000 | 0.939093 | 0.161972 | 2.10065 |
| 10 | 7470001 | 7740000 | 1.468895 | 0.084919 | 2.45512 |
| 10 | 17390001 | 11090000 | 0.882414 | 0.113632 | 2.166615 |
| 11 | 12810001 | 12990000 | 1.270047 | 0.12655 | 2.21527 |
| 11 | 14250001 | 14360000 | 0.913896 | 0.090853 | 2.07685 |
| 12 | 820001 | 1030000 | 1.239679 | 0.097902 | 2.19484 |
| 12 | 1420001 | 1530000 | 0.919749 | 0.107672 | 2.177515 |
| 12 | 3570001 | 3830000 | 1.851305 | 0.1869 | 2.24176 |
| 12 | 5540001 | 5640000 | 0.857809 | 0.108591 | 2.30155 |
| 12 | 10050001 | 10190000 | 0.911106 | 0.109191 | 2.13363 |
| 12 | 11540001 | 11710000 | 1.133051 | 0.108673 | 2.17128 |
| 12 | 12510001 | 12700000 | 1.474365 | 0.080874 | 1.99779 |
| 12 | 13570001 | 13700000 | 0.894359 | 0.102452 | 1.99857 |
| 12 | 19370001 | 19660000 | 1.046025 | 0.12572 | 2.07062 |
| 13 | 2760001 | 2920000 | 0.960815 | 0.076105 | 1.99629 |
| 13 | 11030001 | 11220000 | 0.94373 | 0.091257 | 2.06601 |
| 13 | 14680001 | 14830000 | 0.957303 | 0.088864 | 2.29235 |
| 13 | 16140001 | 16280000 | 0.855548 | 0.066884 | 1.96893 |
| 14 | 8220001 | 8360000 | 1.20085 | 0.111887 | 2.081 |
| 14 | 8730001 | 8900000 | 1.349129 | 0.120616 | 2.15054 |
| 14 | 8980001 | 9120000 | 1.013287 | 0.115617 | 3.387074 |
| 14 | 12650001 | 12780000 | 1.18849 | 0.115796 | 1.98818 |
| 15 | 2230001 | 2370000 | 0.876531 | 0.12666 | 1.97687 |
| 15 | 3510001 | 3650000 | 0.976931 | 0.107665 | 2.09511 |
| 15 | 9870001 | 9970000 | 1.030176 | 0.083215 | 1.96086 |
| 15 | 11080001 | 11220000 | 2.059701 | 0.122353 | 2.15369 |
| 17 | 1010001 | 1200000 | 1.141162 | 0.117372 | 2.49065 |
| 17 | 2520001 | 2700000 | 1.058224 | 0.118933 | 2.2017 |
| 17 | 3670001 | 680000 | 1.112675 | 0.087068 | 2.07083 |
| 18 | 690001 | 790000 | 0.871369 | 0.073848 | 2.11026 |
| 18 | 1190001 | 1340000 | 0.906073 | 0.086215 | 2.00644 |
| 18 | 1370001 | 1640000 | 1.185538 | 0.11346 | 2.247434 |
| 18 | 1960001 | 2120000 | 1.221226 | 0.065664 | 2.100107 |
| 18 | 7020001 | 7130000 | 1.231108 | 0.081341 | 2.11105 |
| 19 | 3650001 | 3820000 | 1.078992 | 0.089738 | 1.96964 |
| 19 | 4490001 | 4670000 | 1.123447 | 0.108918 | 1.97055 |
| 19 | 7310001 | 7430000 | 1.79938 | 0.107385 | 2.079958 |
| 20 | 7780001 | 7920000 | 0.868481 | 0.11221 | 2.173845 |
| 20 | 11890001 | 12160000 | 1.196265 | 0.146146 | 2.080081 |
| 20 | 12710001 | 12870000 | 1.207225 | 0.127179 | 2.39436 |
| 20 | 13020001 | 13240000 | 1.124254 | 0.110425 | 2.033812 |
| 22 | 2570001 | 2710000 | 2.108321 | 0.100715 | 2.05584 |
| 23 | 1830001 | 2000000 | 0.909474 | 0.106547 | 2.23675 |
| 24 | 5570001 | 6030000 | 1.0674 | 0.094916 | 2.41299 |
| 25 | 1330001 | 1520000 | 1.227709 | 0.158663 | 2.20792 |
| 25 | 2430001 | 2650000 | 1.123802 | 0.116751 | 2.00608 |
| 26 | 180001 | 350000 | 3.721244 | 0.139387 | 2.26081 |
| 26 | 1220001 | 1370000 | 4.334661 | 0.207854 | 2.24502 |
| 26 | 2470001 | 1780000 | 0.961439 | 0.12796 | 2.16886 |
| 27 | 4210001 | 4330000 | 1.768227 | 0.202737 | 2.04258 |
| 27 | 4350001 | 4480000 | 1.057151 | 0.101825 | 2.04258 |
| 27 | 4750001 | 4930000 | 1.927256 | 0.137539 | 1.96553 |
| 28 | 820001 | 920000 | 1.120834 | 0.117309 | 2.1309 |
| 28 | 1140001 | 1470000 | 1.243095 | 0.093739 | 2.13852 |
| 33 | 1260001 | 1450000 | 0.84235 | 0.095588 | 2.01021 |
| 33 | 1520001 | 1620000 | 2.537658 | 0.124287 | 2.01021 |
| Z | 3340001 | 3440000 | 0.865412 | 0.109333 | 2.00334 |
| Z | 3450001 | 3570000 | 1.052417 | 0.104977 | 2.00334 |
| Z | 4380001 | 4530000 | 0.97132 | 0.087816 | 2.13607 |
| Z | 14430001 | 14710000 | 1.799934 | 0.08844 | 1.97076 |
| Z | 16210001 | 16390000 | 1.674928 | 0.05718 | 2.1143 |
| Z | 25460001 | 26010000 | 1.666005 | 0.191369 | 2.648177 |
| Z | 26210001 | 26450000 | 1.716793 | 0.095761 | 2.176267 |
| Z | 27920001 | 28030000 | 1.039573 | 0.080874 | 2.1596 |
| Z | 29060001 | 29440000 | 1.719351 | 0.094321 | 2.204259 |
| Z | 31010001 | 31220000 | 1.1646 | 0.07305 | 1.98191 |
| Z | 38230001 | 38400000 | 2.334183 | 0.140443 | 2.02267 |
| Z | 39290001 | 39430000 | 3.11135 | 0.072573 | 2.23038 |
| Z | 39710001 | 39900000 | 1.664412 | 0.09876 | 2.26845 |
| Z | 39960001 | 40100000 | 0.91314 | 0.062211 | 3.19997 |
| Z | 62020001 | 62210000 | 1.909515 | 0.079297 | 2.9491 |
| Z | 62800001 | 62990000 | 1.47241 | 0.093913 | 2.10644 |
| Z | 63450001 | 63630000 | 1.48251 | 0.08005 | 2.035 |
| Z | 66890001 | 67140000 | 1.3066 | 0.170052 | 2.13861 |
| Z | 67210001 | 67320000 | 3.089293 | 0.135031 | 2.602075 |
| Z | 68250001 | 68430000 | 4.078963 | 0.142497 | 2.01739 |
| Z | 69960001 | 70190000 | 1.981724 | 0.14363 | 2.06138 |
| Z | 75450001 | 75760000 | 1.963044 | 0.107594 | 2.06105 |
| Z | 76740001 | 76860000 | 0.900040066 | 0.104735 | 3.12438 |

Baier Yellow Chicken

| Chr | Region | | Log2Pi | F_ST_ | XP-EHH |
| --- | --- | --- | --- | --- | --- |
| 1 | 60110001 | 60300000 | 0.955205082 | 0.1089727 | 2.135654 |
| 1 | 67550001 | 67740000 | 1.1269542 | 0.0916382 | 2.19635 |
| 1 | 68000001 | 68430000 | 1.124254484 | 0.06923396 | 2.1667384 |
| 1 | 68450001 | 68600000 | 1.121324685 | 0.057991033 | 2.595175 |
| 1 | 100560001 | 100720000 | 0.900021343 | 0.08891086 | 2.09946 |
| 1 | 125530001 | 125630000 | 0.853845825 | 0.0846784 | 1.9958 |
| 1 | 133550001 | 133670000 | 0.973518342 | 0.0755698 | 2.19462 |
| 1 | 136310001 | 136430000 | 1.048678952 | 0.101886667 | 2.56572 |
| 1 | 140160001 | 140260000 | 0.878147421 | 0.0843149 | 2.08108 |
| 1 | 140370001 | 140480000 | 0.919586637 | 0.1518415 | 2.33698 |
| 1 | 141650001 | 141750000 | 0.856479979 | 0.0907836 | 2.11787 |
| 1 | 146760001 | 147040000 | 1.24312721 | 0.073607331 | 2.20611 |
| 1 | 148500001 | 148670000 | 1.078012558 | 0.100474 | 2.172201429 |
| 1 | 150310001 | 150540000 | 0.929935441 | 0.0918194 | 2.07268 |
| 1 | 154230001 | 154380000 | 0.997277565 | 0.09064505 | 2.11582 |
| 1 | 163800001 | 163900000 | 0.864389827 | 0.0689522 | 2.30091 |
| 1 | 164320001 | 164460000 | 1.235191468 | 0.086832 | 2.106434 |
| 1 | 174730001 | 174860000 | 1.017366882 | 0.095732525 | 1.9728925 |
| 1 | 176210001 | 176320000 | 0.973945687 | 0.1212375 | 2.06359 |
| 1 | 177470001 | 177620000 | 0.920125907 | 0.0983779 | 2.285648333 |
| 1 | 191280001 | 191400000 | 0.923302046 | 0.081686367 | 2.33801 |
| 1 | 191560001 | 191670000 | 0.86904112 | 0.129999 | 2.157575 |
| 1 | 195530001 | 195700000 | 0.881117395 | 0.083007229 | 1.96197 |
| 2 | 25110001 | 25220000 | 0.931107649 | 0.0828138 | 2.09457 |
| 2 | 26130001 | 26320000 | 1.866927234 | 0.11590994 | 2.05811 |
| 2 | 30940001 | 31090000 | 0.898926172 | 0.100847167 | 2.231133333 |
| 2 | 66190001 | 66290000 | 0.850858031 | 0.080065 | 2.32637 |
| 2 | 91880001 | 92110000 | 1.047009435 | 0.081143758 | 2.21648 |
| 2 | 92340001 | 92450000 | 0.881395716 | 0.09257755 | 2.38129 |
| 2 | 92660001 | 92860000 | 0.98437588 | 0.112209091 | 2.277556364 |
| 2 | 93440001 | 93580000 | 0.929021405 | 0.102437 | 2.1779 |
| 2 | 94470001 | 94600000 | 0.947003699 | 0.0853734 | 2.052023333 |
| 2 | 95520001 | 95620000 | 0.930991092 | 0.128278 | 2.03724 |
| 2 | 100140001 | 100660000 | 1.097683992 | 0.097804134 | 2.553925625 |
| 2 | 100790001 | 100980000 | 1.064425284 | 0.09728905 | 2.344383333 |
| 2 | 101730001 | 101870000 | 1.192173341 | 0.07967458 | 2.32854 |
| 2 | 101940001 | 102080000 | 1.044176104 | 0.09366818 | 2.35454 |
| 2 | 141320001 | 141500000 | 1.34914953 | 0.1195978 | 2.37084 |
| 2 | 143700001 | 143870000 | 1.060133126 | 0.083250938 | 2.13931 |
| 3 | 20001 | 200000 | 1.330199814 | 0.129003222 | 2.29418 |
| 3 | 2000001 | 2180000 | 1.299850728 | 0.112820778 | 2.32559 |
| 3 | 3740001 | 3840000 | 9.023321231 | 0.0864198 | 2.012 |
| 3 | 27600001 | 27720000 | 0.883433091 | 0.0757914 | 1.96647 |
| 3 | 36740001 | 37090000 | 2.353627365 | 0.164050496 | 2.468728846 |
| 3 | 41610001 | 41760000 | 1.421999401 | 0.0771243 | 2.28262 |
| 3 | 41890001 | 41990000 | 1.159987988 | 0.078306 | 2.28262 |
| 3 | 50350001 | 50660000 | 1.814019833 | 0.121778545 | 2.345351818 |
| 3 | 55840001 | 55970000 | 0.885328577 | 0.088807133 | 2.06348 |
| 3 | 63610001 | 63730000 | 0.907442054 | 0.0974081 | 1.98351 |
| 3 | 70030001 | 70150000 | 1.05030929 | 0.145930667 | 2.30486 |
| 3 | 79740001 | 79930000 | 1.268393296 | 0.08862114 | 2.29219 |
| 3 | 79980001 | 80350000 | 1.032872792 | 0.08725785 | 2.63164 |
| 3 | 84440001 | 84640000 | 0.934111593 | 0.097854844 | 2.11003 |
| 3 | 93380001 | 93560000 | 0.882487098 | 0.0832308 | 2.4085 |
| 3 | 102190001 | 102290000 | 0.89443854 | 0.0779998 | 2.08508 |
| 3 | 104900001 | 105070000 | 3.166637789 | 0.173529 | 2.160315 |
| 3 | 108550001 | 108740000 | 1.069666781 | 0.10906335 | 1.99691 |
| 4 | 22730001 | 22930000 | 1.096857957 | 0.103631482 | 2.09304 |
| 4 | 36280001 | 36500000 | 1.276342764 | 0.0734567 | 2.19135 |
| 4 | 36930001 | 37090000 | 1.04408815 | 0.1495915 | 2.07817 |
| 4 | 39420001 | 39520000 | 0.92827278 | 0.0791647 | 2.0668 |
| 4 | 42020001 | 42200000 | 1.078423815 | 0.0917054 | 2.0427 |
| 4 | 48440001 | 48570000 | 0.947900392 | 0.10944 | 1.96515 |
| 4 | 60860001 | 60960000 | 0.859610003 | 0.0677127 | 3.13738 |
| 4 | 62320001 | 62430000 | 0.866109033 | 0.0706126 | 2.0029 |
| 4 | 63480001 | 63620000 | 0.947845619 | 0.06874625 | 2.07541 |
| 4 | 63720001 | 63980000 | 1.163996575 | 0.071544641 | 2.368886471 |
| 4 | 64380001 | 64580000 | 1.492835694 | 0.113876782 | 2.00572 |
| 4 | 65650001 | 65810000 | 1.220234151 | 0.101314486 | 2.669934286 |
| 4 | 67620001 | 67980000 | 2.980083352 | 0.176355059 | 2.213518148 |
| 4 | 71660001 | 71790000 | 0.91564644 | 0.0839765 | 3.9757425 |
| 4 | 71950001 | 72100000 | 1.555687685 | 0.0704207 | 2.933608333 |
| 4 | 79230001 | 79360000 | 0.925210932 | 0.08961065 | 2.12502 |
| 5 | 10001 | 120000 | 2.031508098 | 0.126116 | 2.12064 |
| 5 | 1510001 | 1620000 | 1.015610463 | 0.105056 | 2.09854 |
| 5 | 4260001 | 4510000 | 1.173751526 | 0.10455905 | 2.11085 |
| 5 | 5180001 | 5280000 | 0.954722637 | 0.0789613 | 2.19218 |
| 5 | 5420001 | 5560000 | 1.042583838 | 0.128619 | 2.431866 |
| 5 | 20790001 | 21000000 | 1.527144823 | 0.116271117 | 2.00485 |
| 5 | 21160001 | 21260000 | 0.88585751 | 0.105511 | 2.1561 |
| 5 | 23570001 | 23670000 | 1.001449139 | 0.0805668 | 2.10666 |
| 5 | 35910001 | 36070000 | 0.893692778 | 0.0755162 | 2.2573 |
| 5 | 38260001 | 38360000 | 1.190931515 | 0.1002 | 2.23678 |
| 5 | 44020001 | 44180000 | 1.419955722 | 0.073211571 | 2.008725714 |
| 5 | 48900001 | 49060000 | 1.866917394 | 0.0924173 | 2.00615 |
| 5 | 54970001 | 55080000 | 2.3257364 | 0.164795 | 2.0098 |
| 6 | 10300001 | 10400000 | 0.889696534 | 0.0791283 | 2.28885 |
| 6 | 12530001 | 12720000 | 1.104041083 | 0.09005908 | 2.211846 |
| 6 | 19230001 | 19440000 | 1.628940671 | 0.086054356 | 2.293897778 |
| 6 | 25360001 | 25490000 | 0.887330144 | 0.082899433 | 2.180653333 |
| 7 | 21310001 | 21450000 | 0.968828578 | 0.0797913 | 2.238586 |
| 8 | 13200001 | 13310000 | 1.092800048 | 0.130837 | 1.97919 |
| 9 | 800001 | 960000 | 1.207266944 | 0.116318883 | 2.03552 |
| 9 | 5240001 | 5340000 | 0.889131166 | 0.0781645 | 2.10733 |
| 9 | 6410001 | 6530000 | 1.046606587 | 0.103158 | 2.27684 |
| 9 | 14950001 | 2810000 | 1.076262453 | 0.08730206 | 2.286534 |
| 10 | 6530001 | 6640000 | 0.894850958 | 0.1151885 | 2.06044 |
| 10 | 6800001 | 6920000 | 1.01740084 | 0.099312467 | 2.196843333 |
| 10 | 7100001 | 7210000 | 0.943724629 | 0.0940465 | 2.51248 |
| 10 | 7640001 | 7780000 | 1.068021203 | 0.136494825 | 2.60228 |
| 11 | 560001 | 820000 | 0.912749097 | 0.108658163 | 2.25517 |
| 11 | 1360001 | 1480000 | 1.263572233 | 0.116127733 | 2.359063333 |
| 11 | 10900001 | 11020000 | 1.019658008 | 0.0648506 | 2.12595 |
| 11 | 11130001 | 11260000 | 1.101473429 | 0.0990335 | 2.0829125 |
| 11 | 15650001 | 15760000 | 0.96179176 | 0.110473 | 2.98319 |
| 11 | 17910001 | 18050000 | 0.864678967 | 0.08750366 | 2.0729 |
| 11 | 19120001 | 1190000 | 1.128236376 | 0.090472325 | 2.11639 |
| 12 | 1420001 | 1530000 | 1.243842667 | 0.1086785 | 2.14133 |
| 12 | 2240001 | 2370000 | 1.195287163 | 0.13332 | 2.1852675 |
| 12 | 3230001 | 3340000 | 0.882401932 | 0.1073765 | 2.1588 |
| 12 | 4250001 | 4420000 | 1.114742401 | 0.091162525 | 1.98904 |
| 12 | 5600001 | 5780000 | 1.020335085 | 0.0941722 | 2.15534625 |
| 13 | 3380001 | 3530000 | 1.387729838 | 0.09238895 | 2.06211 |
| 13 | 4410001 | 4570000 | 1.088736399 | 0.0844568 | 2.2669 |
| 13 | 5200001 | 5330000 | 0.942930764 | 0.0821535 | 2.122745 |
| 13 | 6120001 | 6490000 | 0.970240676 | 0.090257874 | 2.010876316 |
| 13 | 14780001 | 14880000 | 0.852245358 | 0.0936615 | 2.16494 |
| 13 | 17560001 | 2800000 | 1.084027516 | 0.096801167 | 2.457546667 |
| 14 | 7160001 | 7270000 | 1.074236443 | 0.109202 | 2.53094 |
| 14 | 8250001 | 8370000 | 0.984945612 | 0.0855341 | 2.05985 |
| 14 | 8730001 | 8930000 | 1.619762071 | 0.137745991 | 2.152473636 |
| 14 | 12610001 | 12780000 | 1.557438496 | 0.136386 | 2.01043 |
| 14 | 14790001 | 4030000 | 0.956873698 | 0.10006925 | 2.09168 |
| 15 | 6070001 | 6190000 | 0.911090627 | 0.090043367 | 2.20842 |
| 17 | 1800001 | 1900000 | 0.866034264 | 0.1019 | 2.33178 |
| 17 | 8550001 | 2020000 | 1.237324077 | 0.0915561 | 2.098055 |
| 18 | 4340001 | 4440000 | 0.937477705 | 0.117368 | 2.3249 |
| 18 | 6420001 | 400000 | 1.450356499 | 0.162861667 | 2.446176667 |
| 20 | 7800001 | 7920000 | 0.934855647 | 0.0803546 | 2.297316667 |
| 20 | 9410001 | 9600000 | 1.04966027 | 0.08784619 | 2.153898 |
| 22 | 2040001 | 2360000 | 1.256378899 | 0.181219053 | 2.20161 |
| 22 | 2460001 | 1750000 | 0.971427697 | 0.1164364 | 2.11802 |
| 23 | 1860001 | 2020000 | 1.001106852 | 0.104056857 | 2.129425714 |
| 23 | 2180001 | 2290000 | 0.983106561 | 0.130037 | 2.32966 |
| 23 | 2430001 | 2530000 | 1.04303099 | 0.0738929 | 2.32966 |
| 23 | 2720001 | 2960000 | 1.062429593 | 0.107091247 | 2.135128667 |
| 26 | 1430001 | 1530000 | 0.872896627 | 0.105108 | 2.09204 |
| 26 | 4500001 | 4620000 | 1.156485421 | 0.0581523 | 2.32208 |
| 27 | 1640001 | 1790000 | 2.300012799 | 0.209869167 | 2.17898 |
| 28 | 1140001 | 1340000 | 1.82431785 | 0.107107425 | 2.00781 |
| 28 | 1350001 | 1510000 | 1.221233641 | 0.0969204 | 2.00781 |
| 28 | 2430001 | 2590000 | 0.922229338 | 0.1105672 | 2.10312 |
| 31 | 1 | 110000 | 3.388596565 | 0.255705 | 22.9769 |
| Z | 26850001 | 27130000 | 1.658477728 | 0.121125158 | 2.38103 |
| Z | 39230001 | 39430000 | 1.9533073 | 0.219184818 | 2.12788 |
| Z | 39710001 | 39810000 | 1.048585852 | 0.0930906 | 2.41108 |
| Z | 41680001 | 41830000 | 1.071877962 | 0.1074174 | 2.13451 |
| Z | 41840001 | 42010000 | 3.049238778 | 0.133356 | 2.25072625 |
| Z | 42460001 | 43010000 | 2.023414774 | 0.147688686 | 2.1041 |
| Z | 45560001 | 45860000 | 1.682383218 | 0.120461724 | 1.98979 |
| Z | 47540001 | 47660000 | 1.769965335 | 0.089123 | 2.20773 |
| Z | 58170001 | 58280000 | 0.912278751 | 0.0698125 | 2.35896 |
| Z | 58350001 | 58550000 | 1.577264765 | 0.107414745 | 2.35896 |
| Z | 59180001 | 59360000 | 1.51067676 | 0.127480778 | 2.03903 |
| Z | 60450001 | 60560000 | 0.986163239 | 0.1056945 | 2.09493 |
| Z | 73030001 | 73130000 | 1.597748566 | 0.218769 | 2.22502 |
| Z | 73160001 | 73330000 | 4.442738133 | 0.122611625 | 2.22502 |
| Z | 75420001 | 75760000 | 2.807264819 | 0.225397 | 1.99655 |
| Z | 77520001 | 77730000 | 1.955382624 | 0.161215909 | 2.10459 |
| Z | 78810001 | 78910000 | 1.105285411 | 0.0623566 | 2.47703 |
| Z | 80560001 | 80680000 | 0.932225969 | 0.117967 | 2.54481 |
